# Expression profiles of east–west highly differentiated genes in Uyghur genomes

**DOI:** 10.1093/nsr/nwad077

**Published:** 2023-03-21

**Authors:** Zhilin Ning, Xinjiang Tan, Yuan Yuan, Ke Huang, Yuwen Pan, Lei Tian, Yan Lu, Xiaoji Wang, Ruicheng Qi, Dongsheng Lu, Yajun Yang, Yaqun Guan, Dolikun Mamatyusupu, Shuhua Xu

**Affiliations:** Key Laboratory of Computational Biology, Shanghai Institute of Nutrition and Health, University of Chinese Academy of Sciences, Chinese Academy of Sciences, Shanghai 200031, China; Key Laboratory of Computational Biology, Shanghai Institute of Nutrition and Health, University of Chinese Academy of Sciences, Chinese Academy of Sciences, Shanghai 200031, China; Key Laboratory of Computational Biology, Shanghai Institute of Nutrition and Health, University of Chinese Academy of Sciences, Chinese Academy of Sciences, Shanghai 200031, China; Key Laboratory of Computational Biology, Shanghai Institute of Nutrition and Health, University of Chinese Academy of Sciences, Chinese Academy of Sciences, Shanghai 200031, China; School of Life Science and Technology, Shanghai Tech University, Shanghai 201210, China; Key Laboratory of Computational Biology, Shanghai Institute of Nutrition and Health, University of Chinese Academy of Sciences, Chinese Academy of Sciences, Shanghai 200031, China; Key Laboratory of Computational Biology, Shanghai Institute of Nutrition and Health, University of Chinese Academy of Sciences, Chinese Academy of Sciences, Shanghai 200031, China; State Key Laboratory of Genetic Engineering, Center for Evolutionary Biology, Collaborative Innovation Center of Genetics and Development, School of Life Sciences, Fudan University, Shanghai 200438, China; Key Laboratory of Computational Biology, Shanghai Institute of Nutrition and Health, University of Chinese Academy of Sciences, Chinese Academy of Sciences, Shanghai 200031, China; Key Laboratory of Computational Biology, Shanghai Institute of Nutrition and Health, University of Chinese Academy of Sciences, Chinese Academy of Sciences, Shanghai 200031, China; Key Laboratory of Computational Biology, Shanghai Institute of Nutrition and Health, University of Chinese Academy of Sciences, Chinese Academy of Sciences, Shanghai 200031, China; State Key Laboratory of Genetic Engineering, Center for Evolutionary Biology, Collaborative Innovation Center of Genetics and Development, School of Life Sciences, Fudan University, Shanghai 200438, China; Department of Biochemistry and Molecular Biology, Preclinical Medicine College, Xinjiang Medical University, Urumqi 830011, China; College of the Life Sciences and Technology, Xinjiang University, Urumqi 830046, China; State Key Laboratory of Genetic Engineering, Center for Evolutionary Biology, Collaborative Innovation Center of Genetics and Development, School of Life Sciences, Fudan University, Shanghai 200438, China; Human Phenome Institute, Zhangjiang Fudan International Innovation Center, and Ministry of Education Key Laboratory of Contemporary Anthropology, Fudan University, Shanghai 201203, China; Department of Liver Surgery and Transplantation, Liver Cancer Institute, Zhongshan Hospital, Fudan University, Shanghai 200032, China

**Keywords:** gene expression, RNASeq, Xinjiang's Uyghurs, next-generation sequencing

## Abstract

It remains unknown and debatable how European-Asian–differentiated alleles affect individual phenotypes. Here, we made the first effort to analyze the expression profiles of highly differentiated genes with eastern and western origins in 90 Uyghurs using whole-genome (30× to 60×) and transcriptome data. We screened 921 872 east–west highly differentiated genetic variants, of which ∼4.32% were expression quantitative trait loci (eQTLs), ∼0.12% were alternative splicing quantitative trait loci (sQTLs), and ∼0.12% showed allele-specific expression (ASE). The 8305 highly differentiated eQTLs of strong effects appear to have undergone natural selection, associated with immunity and metabolism. European-origin alleles tend to be more biasedly expressed; highly differentiated ASEs were enriched in diabetes-associated genes, likely affecting the diabetes susceptibility in the Uyghurs. We proposed an admixture-induced expression model to dissect the highly differentiated expression profiles. We provide new insights into the genetic basis of phenotypic differentiation between Western and Eastern populations, advancing our understanding of the impact of genetic admixture.

## Teaser

East-west differentiated gene expression profiles.

## INTRODUCTION

The mechanisms and driving forces of phenotypic diversity remain a mystery despite the fact that more than two decades have passed since the studies entered the genomic era. One of the major challenges could be that genes associated with racial differentiation or speciation are often non-essential [1]. In humans, the genetic and phenotypic differences between East Asian (EAS) and West European populations have long been studied. Large numbers of population genetic studies and genome-wide association studies (GWAS) of EAS and West European populations have revealed population differences and have provided insights into various clinical applications concerning disease-associated alleles in ethnic groups of different ancestry backgrounds [2–4]. Most GWAS have been conducted in populations of European ancestry, and the genetic basis of phenotype differentiation between European (EUR) and EAS populations remains largely unknown. It is challenging to address this question due to the many confounding factors and stochastic biases, such as population genetic architecture, living habits, technical variation and sample size, that are present in comparative analyses between different ethnic groups [5,6]. Recently admixed Eurasian populations, such as the Uyghurs, provide a unique opportunity to investigate the impact of genetic variation on a finer scale in a single population with both western and eastern ancestry [7–9]. Long-term admixture has resulted in the Xinjiang Uyghur (XJU) population harboring relatively uniformly distributed ancestry across individual genomes and provides high-resolution gene–gene and allele–allele interaction detection. In particular, the combination of the relatively high-frequency of alleles of distinct ancestral origin greatly increases the statistical power in gene regulation analyses. Furthermore, combinations of alleles of distinct ancestral origins facilitate investigation of hitchhiking under positive Darwinian selection as well as negative selection on the human genome [10,11].

Taking advantage of the unique genomic and transcriptomic architectures of the Uyghurs living in the XJU Autonomous Region, we conducted the first study of blood transcriptomics in this Eurasian admixed population via whole-genome sequencing of DNA and RNA from whole blood. In this study, we concentrated on the influence of highly differentiated genetic variants on the transcriptomic patterns between Eastern and Western populations. Accordingly, we identified highly differentiated genetic variants and genes from the east and west, analyzed their putative functional effects, and explored their evolutionary and medical implications.

## RESULTS

### Identifying highly differentiated genes between the east and west

We performed a genome-wide analysis of genetic differentiation between EAS and EUR based on the 1000 Genomes Project data [3,4,12] (KGP, Materials and Methods). We screened 2 346 869 single nucleotide variants (SNVs) and identified 921 872 highly differentiated SNVs (HDSs, defined as SNVs with fixation index ${F}_{ST} > 0.2$) covering 17 151 genes (Materials and Methods). HDSs were enriched in epigenetic regulation regions, including weakly repressed polycomb regions and quiescent/low chromatin features (*P* < 10^−16^, Supplementay Fig. S9). Approximately 0.77% of HDSs (7059) were identified as lead SNVs in previous GWASs (the NHGRI-EBI GWAS Catalog) [13]. In particular, these GWAS-overlapped HDSs were enriched in associated traits or diseases such as inflammatory bowel disease (IBD), hair color, body mass index, neuroticism, and mean arterial pressure (*P <* 0.05, Fisher's Exact Test, Materials and Methods and Supplementay Fig. S10). Among HDS-covered genes, the most significant enriched trait was diastolic blood pressure; however, those genes were related to multiple pathways, including metabolism, immunity and inflammatory associated pathways (Supplementay Figs S11 and S12).

At the gene level, we identified 1567 highly differentiated genes (HDGs, Materials and Methods, Supplementay Fig. S13). The top 10 traits/diseases associated with HDGs were diastolic blood pressure, the total duration of ventricular activation and recovery (QT interval), high-density lipoprotein cholesterol, platelet count, nonsyndromic cleft lip with cleft palate, coronary heart disease, aspartate aminotransferase levels, psoriasis, systolic blood pressure, and chronic inflammatory diseases (Supplementay Fig. S14). Notably, blood pressure and inflammatory diseases were enriched with genes classified as HDSs and HDGs, respectively. Previous studies have shown that EAS and Western European populations differed considerably in the burden of hypertension [14] and inflammatory diseases such as IBD [15,16] and atopic dermatitis [17–21]. Compared with genome-wide SNVs, the HDSs showed significant functional potential Combined Annotation Dependent Depletion (CADD, *P* < 10^−16^, Supplementay Fig. S15), suggesting that they are important components of the genetic basis of phenotypic differences between EAS and EUR populations.

### Identifying eQTLs from HDSs

To investigate the influence of HDSs on gene expression patterns, we further identified expression quantitative trait loci (eQTLs) and alternative splicing quantitative trait loci (sQTLs) by analyzing the genetic regulation of HDSs. The results showed that 39 854 HDSs were eQTLs (HDS-eQTLs, 4.32%), 1063 HDSs were sQTLs (HDS-sQTLs, 0.12%), and 499 overlapped between the two categories. Moreover, 294 HDGs were eGenes (HDG-eGenes, 18.77%), 87 HDGs were sGenes (HDG-sGenes, 5.56%), and 58 overlapped between the two categories (Materials and Methods). We found that HDSs were more likely to be eQTLs (Fisher's Exact Test, *P* < 10^−16^, odds ratio (OR) = 3.24) but less likely to be sQTLs (Fisher's Exact Test, *P* < 10^−16^, OR = 0.24). At the genome-wide level, eQTLs showed larger ancestral genetic differences than that of a set of random variants (non-eQTLs), and sQTLs showed smaller ancestral genetic differences than that of a set of random variants (non-sQTLs, one-tailed *t* test, *P* < 10^−16^; Materials and Methods and Supplementay Fig. S16). Notably, the number of eQTLs in HDSs was positively correlated with the ancestral genetic difference between EAS and EUR (*P* < 10^−16^), while the number of sQTLs in HDSs was negatively correlated with the ancestral genetic difference between EAS and EUR populations (linear regression model: *P* < 10^−16^, Supplementay Figs S17 and S18).

The regulatory regions of HDS-eQTLs and HDS-sQTLs were significantly enriched in regions associated with certain regulatory elements, such as active transcription start sites (TSS) and transcription at the 5′ and 3′ regions (*P* < 10^−16^, Supplementay Figs S19 and S20). HDS-eQTLs were also enriched in GWAS-loci (Supplementay Fig. S21). Some notable examples included macular thickness and blood traits such as neutrophil count, white blood cell count, myeloid white cell count, sum neutrophil eosinophil counts and granulocyte count. In contrast, HDS-sQTLs were significantly enriched in GWAS-loci associated with traits of systolic and diastolic blood pressure traits (Supplementay Fig. S22).

To evaluate the validity of HDS-QTLs, we compared our results with public datasets of Western and Eastern populations. First, we evaluated the replication of HDS-QTLs with European QTLs found in the Lymphoblastoid Cell Lines (LCLs) [Genetic European Variation in Health and Disease (GEUVADIS)] and whole blood (the Genotype-Tissue Expression; GTEx) [22]. For HDS-eQTLs, the replication rates were 76.28% in GEUVADIS and 68.13% in GTEx; for sQTLs, the replication rates were 71.59% in GEUVADIS and 65.57% in GTEx. Next, we compared HDS-QTLs with eQTLs detected in healthy Japanese volunteers’ immune cells [23]. For HDS-eQTLs, the average replication rate was ∼45% in different types of immune cells, consistent with the eQTL replication rates between whole blood and immune cells reported in the latter study. Therefore, these results indicated a considerable degree of gene expression regulation shared among the XJU, Eastern and Western populations.

### Identifying ASE from HDSs

To further estimate the relative contribution of each *cis*-regulatory variant in HDSs, we meticulously analyszed allele-specific expression (ASE). We identified 1067 cases of ASE (0.12%, HDS-ASE) in HDSs in 690 genes (HDS-aseGenes) accounting for 12% (1067/8913) of the ASEs detected in the XJU and 25% (690/2811) of the aseGenes detected in XJU (Materials and Methods). Per individual, there were ∼32 HDS-ASEs (SD = 10.82), accounting for ∼11%, and ∼0.001% of these were heterozygotes (Table S3). In total, ∼37% of HDS-ASE overlapped with HDS-eQTLs; only ∼1% overlapped with HDS-sQTLs, and merely ∼8% (50/690) of HDS-aseGenes overlapped with HDG. Thus, it is more likely for an HDS to be an ASE site (Fisher's Exact Test, *P* < 10^−16^, OR = 2.81). Replication analysis of GTEx (whole blood) data confirmed ∼95.41% of the HDS-ASE sites, reflecting a relatively consistent regulatory role of HDS-ASE among populations with different ancestry.

Similar to HDS-QTLs, HDS-ASEs were most significantly enriched in regulatory regions of transcription at the 5′ and 3′ ends of genes, in genic enhancers, in strong transcription sites, active TSSs and regions flanking active TSSs (*P* < 10^−16^, Supplementay Fig. S23). In addition, the HDS-ASEs were closely related to blood traits such as neutrophil count, sum neutrophil and eosinophil counts, sum basophil neutrophil counts, myeloid white cell counts, granulocyte counts and hemoglobin concentration (*P* < 0.05, Supplementay Fig. S24). For HDS-aseGenes, these showed evidence of being related to osteoclast differentiation (*P* < 10^−16^), nature killer cell-mediated cytotoxicity (*P =* 0.015, Supplementay Fig. S25), diastolic blood pressure (*P* < 0.05) and chronic inflammatory diseases (*P* < 0.05, Supplementay Fig. S26). The enrichment analysis indicated the potential regulatory effects of HDSs on certain phenotypes, such as blood traits and blood pressure.

**Figure 1.**
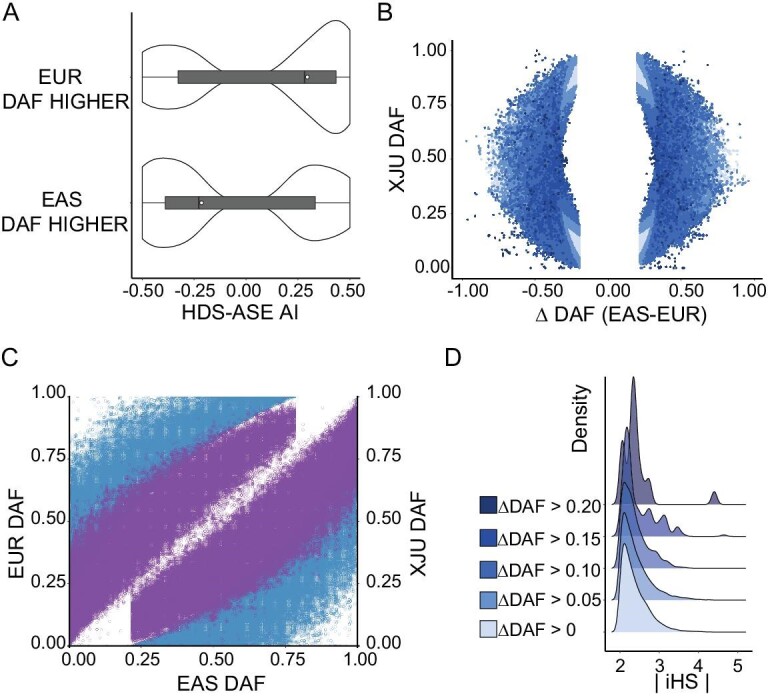
Properties of HDSs detected in XJU. (A) Comparison of the AI of HDS-ASE. The HDS-ASE were divided into two groups by derived allele frequency (DAF). If the allele frequency of the derived allele of ASE was higher in EUR, then the allele would be regarded as a western origin allele with a high probability (labeled as EUR DAF HIGHER in the figure). If the allele frequency of the derived allele of ASE was higher in EAS, the allele would be regarded as an eastern origin allele with a high probability (labeled as EAS DAF HIGHER in the figure). The x-axis indicates the AI levels. The ‘+’ indicates that the derived alleles were highly expressed, and the ‘−’ indicates that the ancestral alleles were highly expressed. (B) DAF distribution of HDSs. The x-axis indicates the frequency differences between EAS and EUR ($| {\Delta {\rm{DAF}}} |$), and the y-axis indicates the DAF in XJU. Each dot reflects one SNP. The colors of dots denote the frequency deviation degree measured as$| {{\rm{\ expected\ DAF}} - {\rm{observed\ DAF}}} |$ (Materials and Methods). (C) HDS DAF distribution in XJU with its ancestral source populations. Each dot indicates one SNP in HDSs. The colors of the dots indicate the mapping to the y-axis. The color blue indicates the allele frequency distribution between EAS and EUR, and the color purple indicates the allele frequency distribution between EAS and XJU. (D) A density plot of HDS integrated haplotype score (iHS) with R package ‘ggridges’ [68]. (See online supplementary material for a colour version of this figure).

On a genome-wide level, we also observed a positive correlation between the percentage of ASEs and the ancestral genetic difference that was similar to the pattern observed for eQTLs (Supplementay Fig. S27). In contrast, ASE showed fewer ancestral genetic differences than a set of random variants (non-ASE, one-tailed *t* test, *P* < 10^−16^, Materials and Methods and Supplementay Fig. S28). We sought to determine how highly differentiated alleles between east and west were expressed when they were located at the same loci. In XJU, when the frequency of one allele was higher in EUR than that in EAS ($\Delta DA{F}_{| {EAS - EUR} |} > 0.20$), the allele was regarded as an allele of western origin; if vice versa, the allele was regarded as an allele of eastern origin. Overall, 91.57% of HDS-ASEs displayed unbalanced expression in alleles with different ancestral genetic backgrounds. Among these, 51.17% of HDS-ASEs of western origin were overexpressed. We compared the degree of imbalance in allele expression [allelic imbalance (AI)] between derived alleles and ancestral alleles and observed that when derived alleles of HDS-ASEs were alleles of western origin, the derived alleles were overexpressed (one-tailed *t* test, *P* < 10^−16^); when derived alleles of HDS-ASE were of eastern origin, the ancestral alleles were overexpressed (one-tailed *t* test, *P* < 10^−16^, Fig. 1A). As a control, we checked the expression profiles of all ASE variants and found that both eastern- and western-derived alleles were overexpressed (one-tailed *t* test, *P* < 10^−16^, Supplementay Fig. S29). Our results suggest that alleles in highly differentiated loci between east and west demonstrate unbalanced expression, and western-ancestry backgrounds tend to demonstrate an increase in the degree of unbalanced expression. One example was rs3213445, a missense variant on *CPT1B.* The *C* allele of this variant was an allele of eastern origin [*C* allele frequency (AF): EAS: 0.39, EUR: 0.06, XJU: 0.26], while the other *T* allele was an allele of western origin. The western allele was overexpressed in eight subjects of XJU (AI ranged from 0.31 to 0.5). This variant was an eQTL in XJU and EAS. It was reported that rs3213445 is nominally associated with triglycerides and low-density lipoprotein-cholesterol levels, and carriers of the rs3213445-*C* variant had significantly higher γ-glutamyl transpeptidase, glutamic oxaloacetic transaminase, and glutamic pyruvate transaminase activities as well as a higher fatty liver index [24–26]. Another example was rs469390, a missense variant on *MX1.* The *G* allele of this variant was an allele of eastern origin (*G* allele frequency: EAS: 0.79, EUR: 0.43, XJU: 0.61), while the *A* allele was of western origin. In XJU, four subjects demonstrated overexpression of the western origin allele. This variant has been reported to be possibly associated with susceptibility to coronavirus disease 2019, and it was suspected that rs469390-*A* expression in the lung might be associated with the high TMPRSS2 expression levels and further associated with the higher susceptibility to the disease [27]. These results suggest that the admixture-induced ancestral origin alleles might increase or decrease the risk of disease in XJU.

The driving force of the abovementioned phenomena could be *cis* genetic regulators or nonsense single-nucleotide polymorphisms (SNPs) where protein-truncating variants cause nonsense-mediated decay (NMD) on ASE loci [28,29]. We found that 822 HDS-ASEs were possibly explained by *cis* genetic regulators, of which 26 619 putative regulatory variants (HDS-aseQTLs) were identified within a +/−100 kb window of HDS-ASE (*P* < 0.05; Materials and Methods), and 293 HDS-ASEs were driven by NMD, of which ∼11% were missense variants and 16% were loss-of-function variants. The remaining HDS-ASE was probably explained by epigenetics or other potential regulatory effects via *trans* variation. As expected, these HDS-aseQTLs were clustered near many regulatory regions, including transcription factors at 5′ and 3′ ends, enhancers, and TSSs, representing eQTL signals of functional importance (Supplementay Fig. S30).

### HDSs underlying natural selection

**Figure 2.**
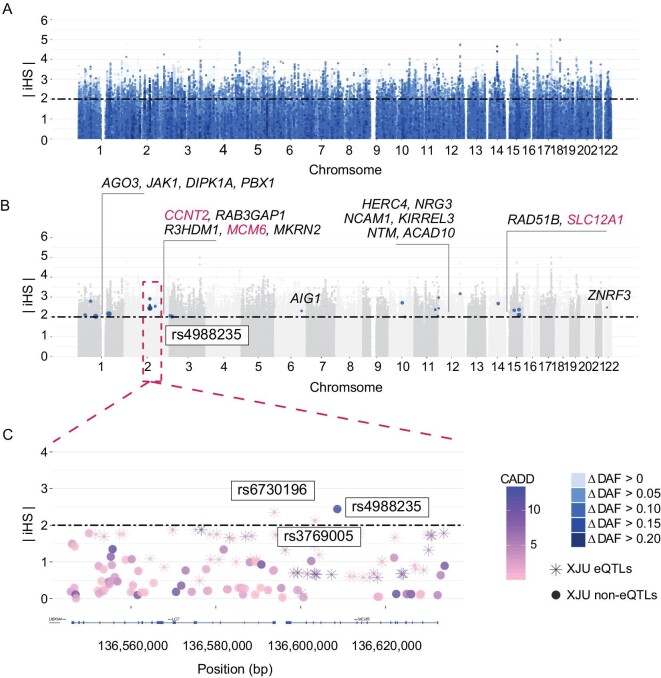
Distribution of iHS in HDSs. (A) Manhattan plots exhibiting the distribution of iHS. Each dot indicates one SNP of HDSs. The x-axis indicated the positions of the SNPs on chromosomes. The y-axis indicates the absolute iHS score. The colors of the dots indicate the degree of frequency deviation in XJU. (B) Distribution of HDSs with high-frequency deviation and potential functions. We labeled the SNP-located genes in the figure. (C) Zoomed-in region of rs4988235. All variants with this region were plotted and coordinated with positions. The genes were labeled by LocusZoom [69]. (See online supplementary material for a colour version of this figure).

To further explore the influence of admixture or selective pressures on the prevalence of these HDS alleles in XJU, we first compared the allele frequencies of HDSs in this population with those in the ancestral source populations, i.e. EAS and EUR. We observed that HDSs were common in at least one of the EAS and EUR, as expected (Fig. 1B). The allele frequency difference between EAS and EUR was highly correlated with the allele frequency of XJU (Fig. 1C), in line with the allele frequency distribution due to admixture [30]. Next, we considered the deviation between the observed and expected allele frequency of HDSs in XJU (${\rm{\Delta }}DA{F}_{XJU}$, Materials and Methods). We found that such deviation existed when HDSs showed a discrepancy in the tendency of XJU allele frequency and allele frequency difference between EAS and EUR. HDSs with a higher degree of deviation indicated evidence of adaptation (Fig. 2A and Supplementay Fig. S31). Furthermore, ∼3.73% of HDSs showed evidence of hard selective sweeps (integrated haplotype scores $| {iHS} |\ > \ 2$). When the degree of deviation was higher, the evidence for harder selective sweeps was stronger (Fig. 1D). Twenty-four HDSs reported by GWAS had a high degree of deviation (${\rm{\Delta }}DA{F}_{XJU} > 0.1$) and a higher probability of reflecting adaptation (Fig. 2B). The most notable case was rs4988235 (Fig. 2C), located upstream of exon 1 of LCT in intron 13 of the *MCM6* gene [31], which has been shown to affect the transcription of enzymes and control the distribution of lactase phenotypes in human populations. The *A* allele of this SNP is associated with lactase persistence and has been shown to increase the LCT gene promoter activity after binding transcription factors [32,33]. The *A* allele in XJU was derived from its western ancestry (AF: EAS: 0, EUR: 0.72, XJU: 0.06), and its frequency in XJU deviated from the expectation (${\rm{\Delta }}DA{F}_{XJU} = 0.30$). This allele shows evidence of strong selection in Europeans [34–36] and has shown signs of adaptation in XJU ($| {iHS} |\ = \ 2.44$). Another two *LCT* SNPs near rs4988235 also showed signs of adaptation. One was rs3769005, which was in strong linkage disequilibrium (LD) with rs4988235 in Europeans [*r*^2^ (coefficient of determination) = 0.72 in Utah residents with Northern and Western European ancestry (CEU) (Materials and Methods)] but not in XJU individuals, possibly due to recombination during admixture (${\rm{\Delta }}DA{F}_{XJU} > 0.15$). Both were eQTLs of the *MCM6* gene in XJU, potentially affecting lactase persistence [37]. This example indicates that admixture brought new alleles and altered the original LD from western ancestry to XJU; some of these alleles were under selection due to living conditions, environmental factors, or consumer habits such as milk intake brought from ancestors during admixture.

**Figure 3.**
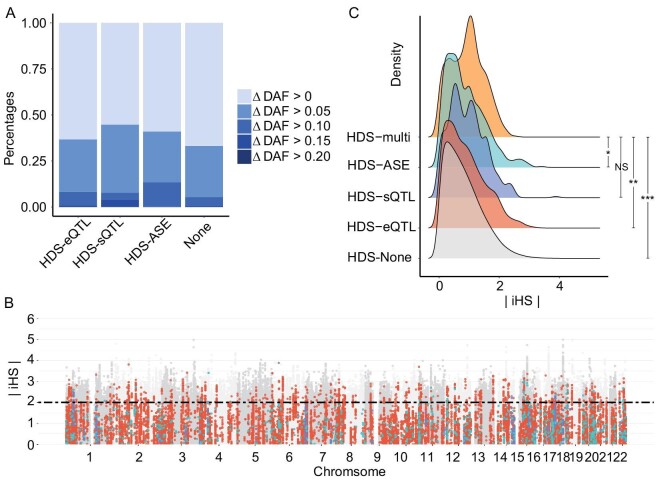
The properties of HDSs with putative functions. (A) A bar plot of frequency deviations of HDSs with putative functions. The x-axis indicates the putative functions of HDSs. The label ‘none’ indicates HDSs without putative functions. The colors indicate the degree of frequency deviation in XJU. (B) Manhattan plots exhibit the distribution of hits with putative functions. Each dot indicates HDSs with putative functions. The x-axis indicated the genetic positions of the SNPs on chromosomes. The y-axis indicates the absolute iHS score. The colors of the dots indicate functional types detected in XJU. The red indicates eQTLs, blue indicates sQTLs, and green indicates ASE loci. (C) Density plot of HDS iHS by the functional groups detected in HDSs. ‘HDS-multi’ indicated HDSs with more than one potential function identified, such as ASE, eQTL, and sQTLs. One-tailed *t* test was applied on $| {i\!H\!S} |$ of HDSs with multiple putative regulatory functions (HDS-multi), and HDSs with single or no putative regulatory functions (HDS-eQTL/HDS-sQTL/HDS-ASE/none). The number of *suggested the significance measured by adjusted *P* value. *P < 0.05, **P < 0.01, ***P < 0.001. NS, not significant. (See online supplementary material for a colour version of this figure).

Additionally, we found that both metrics of conservation (Genomic Evolutionary Rate Profiling, GERP) and predicted functional relevance (CADD) identified significant associations with HDSs conditioned on the degree of deviation (Supplementay Fig. S32), suggesting potential regulatory functions of the HDSs and possible selective pressure. HDSs with putative regulatory functions (HDS-QTLs and HDS-ASE) showed a higher degree of deviation compared with HDSs without putative regulatory functions (Fig. 3A and Supplementay Fig. S33). In total, ∼2% of HDSs had putative regulatory functions that showed evidence of hard selective sweeps (Fig. 3B). We next searched for evidence of natural selection among different types of putative regulators of HDSs. We found that HDSs with multiple putative regulatory functions may have undergone stronger selective pressure compared with HDSs with single putative regulatory functions or HDSs with non-predictive functions (adjusted *P* < 0.05; Fig. 3C). These HDSs were significantly enriched in regulatory regions of transcription at gene 5′ and 3′ ends, strong and weak transcription, genic enhancers, active TSSs, flanking active TSSs and enhancers (*P* < 10^−16^; Supplementay Fig. S34). One outstanding example was rs1047911, a missense variant of the *MRPL53* gene detected as an eQTL and ASE of the *MRPL53* gene. It has been reported that rs1047911 is a GWAS locus of autoimmune thyroid disease (AITD) in Europeans [38], and that female Uyghur individuals are more susceptible to AITD [39]. The potential regulatory function and the evidence for adaptation ($| {iHS} | = \ 2.03$) of this variant suggested an association with increased risk for AITD in XJU.

### HDS-eQTLs with regulatory effects on the immune system, infection and metabolism

**Figure 4.**
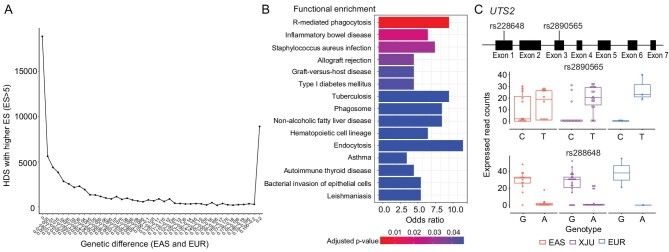
Properties of HDSs with high effects and the example of T2D-susceptible gene. (A) The tendency of the number of eQTLs with ancestral genetic differences. We measured the tendency of the number of all eQTLs having large ES (ES > 5) with an ancestral genetic difference (${F}_{ST}{\rm{\ }}$between EAS and EUR). We divided eQTLs with large ES into 40 blocks by increased ancestral genetic difference (step = 0.005,$\ {F}_{ST}{\rm{\ }}$between EAS and EUR). A SNP is labeled as ‘0’ when the allele difference between ancestral populations is equal to or less than 0; a SNP is labeled as ‘0.2’ when the allele difference between ancestral populations is equal to or larger than 0.2. The y-axis indicates the number of eQTLs. The ancestral genetic difference of XJU SNPs was calculated with the allele frequency of sites in ancestral source populations of XJU. (B) Functional enrichment of HDS-eQTLs with high effects. (C) A typical example of the T2D-susceptible gene *UTS2* in XJU. The schematic diagram of *UTS2* is shown at the top. The bottom box plots show the unbalanced allelic expression levels of rs2890565 and rs228648 in XJU, EAS, and EUR. Each dot represents one sample.

Although there were rare QTLs showing extremely large effect size (ES) and less differentiation between XJU and its ancestral source populations (eQTLs, Pearson correlation, *r* = 0.21, *P* < 10^−16^; sQTLs, Pearson correlation, *r* = 0.61, *P* < 10^−16^; Supplementay Fig. S35), a substantial number of HDS-eQTLs with high ES were observed (ES > 5, HDSE, Fig. 4A). In total, 8305 HDSEs were associated with 335 genes (HDSE-genes). These HDSE-genes were tightly connected in functional networks (Supplementay Fig. S36) that were related to type 1 diabetes mellitus (T1D), nonalcoholic fatty liver disease (NAFLD), tuberculosis (TB), IBD, asthma, AITD, leishmaniasis and other diseases involving bacterial invasion (Fig. 4B), all of which were previously reported in XJU. For example, several major risk factors for T1D were found in Uyghur children [40]; the prevalence of NAFLD was higher in the Uyghur population when compared with the local Han Chinese population in Xinjiang [41,42]. Genetic factors may be involved in the occurrence and development of asthma in XJU [43]; female Uyghur individuals are more susceptible to hypothyroidism and thyroid autoimmune diseases [39], and there were two outbreaks of leishmaniasis disease in Jiashi County, Xinjiang, from 2005 to 2015, although this disease is well-controlled in China [44]. Numerous previous reports support the hypothesis that HDSEs have regulatory effects on transcriptomes, reflecting the influence of the admixture-induced ancestral genetic differences in the immune system, susceptibility to infection, and metabolism in the blood tissue in XJU.

Many HDSE-genes are conserved across species; for example, *FADS2*, reported to encode fatty acid desaturase, was detected with HDS-eQTLs with a large evolutionary conservation score (CADD > 15 and GERP > 4). Six of 335 HDSE-genes, *FADS2, HERC2, DUT, ARHGAP26, CPNE1* and *CTNNA1* were reported to have undergone positive selection in XJU [45]. In addition, ∼5% of HDSEs showed evidence of selective sweeps ($| {iHS} | > 2$).

### HDSs enriched in type 2 diabetes risk genes

We next tested whether type 2 diabetes (T2D) present in XJU was reflected among HDSs. At present, XJU has been reported as having a higher prevalence of diabetes (10.47%) compared with the Han Chinese population (7.36%) [46]. XJU also shows evidence of higher insulin resistance than that of the Han Chinese [47] and Xinjiang Kazak populations [48]. We identified significant enrichment for known T2D risk loci (*P* < 10^−16^) among HDSs (Materials and Methods), reflecting the potential regulation evidence of HDSs on T2D.

The typical T2D-susceptible gene *UTS2* was identified with several HDS-eQTLs, in which two loci, rs2890565 and rs228648, were also identified as ASEs (Fig. 4C). It has been reported that the expression levels of rs2890565-*T* and rs228648-*G* are associated with an insulin-resistant phenotype in the Japanese population [49]. In XJU, risk alleles at the two loci were overexpressed in most of individuals with ASEs, a situation that was potentially driven by an aseQTL, rs664689 (1 : 7 970 092), an intron variant of *UTS2*. The ASE would be driven in heterozygotes for this aseQTL but not in homozygotes with alternative alleles (Supplementay Fig. S37). This SNP of the aseQTL was nearly fixed in the western ancestral source population of XJU (reference allele *C*: AF < 0.02, KGP: EUR; AF = 0.60, KGP: EAS), and thus the reference allele was inferred from eastern ancestors of XJU, and the alternative allele was inferred from western ancestors of XJU. It has been suggested that the admixture brought extra SNPs in XJU and induced potential genetic regulatory effects on gene expression that would also increase the susceptibility to diabetes, thus providing evidence that admixture effects can induce phenotypic variation with potential new driving mechanisms in the admixed group.

In addition, another two T2D-susceptible genes, *KCNQ1* and *HMG20A*, were found to have putative regulatory associations with HDSs. The *KCNQ1* gene was identified as a T2D GWAS gene in the Asian and Uyghur populations [50]. We identified one HDS in *KCNQ1*, rs8234, that was an ASE in XJU and has been shown experimentally to influence *KCNQ1* expression levels [51]. Regarding the *HMG20A* gene, rs952471 was identified as an HDS-ASE. The susceptible allele *C* was highly expressed in XJU. The two ASEs on T2D-susceptible genes suggested the possibility of T2D-regulation through unbalanced allele expression in XJU.

### Local ancestry of HDSs

By inferring and analyzing local ancestry in XJU, we identified 825 genes with expression levels significantly correlated with ancestral source populations; these were regarded as ancestral-like genes in XJU (Materials and Methods). The expression levels of 407 of these genes were positively correlated with the proportions of eastern ancestry (eastern ancestral-like genes), and the expression levels of 409 genes were positively correlated with the proportions of western ancestry (western ancestral-like genes). We identified 31 ancestral-like genes with quantitative trait ancestry segments (eQTAS-genes; Materials and Methods and Table S4). These 31 eQTLAS-genes were related to metabolism and immunity, and almost all of them (28 of 31) were also HDS-eGenes or/and HDS-aseGenes. This confirmed our above deduction that the admixture process had potential regulatory effects on metabolism and immunity in HDSs.

**Figure 5.**
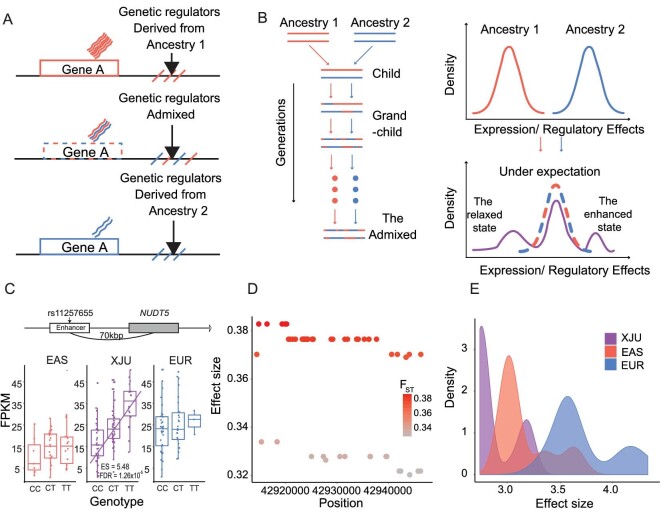
A schematic diagram of the admixture-induced gene expression model with examples. (A) A schematic diagram of ancestral-like expressed genes and the associated regulator. (B) A schematic diagram of the admixture-induced gene expression model. The left part of the Figure illustrates the admixture process, and the right part shows the changes in gene expressions with jointly regulatory effects of genetics and ancestral backgrounds before and after the admixture. In the right part, the distributions in the ancestors and the admixed group indicate the density distributions of the gene expressions or the jointly regulatory effects. The schematic Figure made distribution differences zoomed-in to show the differences clearly. After admixture, the dashed line indicates the expected distribution of the gene expressions or the jointly regulatory effects, and the purple line indicates the observed gene expressions or jointly regulatory effects. (C) An eQTL example in the admixed group-specific state in XJU. The locus, rs7115703, was identified as a candidate enhancement regulator located at 70 bp upstream of *NUDT5.* (D) The ancestral genetic difference distribution of 53 candidate relaxation regulators of the *PEX6* gene in XJU. The genetic differences were measured as$\ {F}_{ST}$ between EAS and EUR. Each dot represents one variant. The x-axis indicates the position of variants across genomes, and the y-axis and the color indicate the value of ${F}_{ST}$. (E) The ES distributions of 53 candidate relaxation regulators of the *PEX6* gene in XJU, EAS, and EUR. The ESs were measured in eQTLs association studies. FDR, false discovery rate; FPKM, fragments per kilobase of transcript per million mapped reads. (See online supplementary material for a colour version of this figure).

Among the 31 eQTAS-genes, those with high expression levels positively correlated with the proportions in eastern ancestry, proportions were always positively associated with segments derived from eastern ancestry, and vice versa. This finding indicated that the admixture-induced genes and their regulatory elements were derived from the same ancestors of the admixed groups. When the combination occurred, the regulatory effects became more moderate than those in ancestral populations (Fig. 5A). One outstanding example was one of the eQTAS-genes, *UTS2*. The expression levels of the gene were positively correlated with the eastern ancestry proportions and were positively associated with several surrounding segments derived from eastern ancestors (Supplementay Fig. S38). Another example was *PGM5* which metabolizes glucose-1-phosphate into glucose-6-phosphate and has potential use as a diagnostic and prognostic biomarker for liver cancer [52]. The expression levels of this gene were positively correlated with the western ancestry proportions and were positively associated with several surrounding segments derived from western ancestry (Supplementay Fig. S39). These results suggested the difference between eastern and western regulatory effects on these genes. A total of 119 HDS-eQTLs with high ES were identified in these associated segments, suggesting that causal variants might exist in, or were highly linked to, our candidate SNPs, confirming our hypothesis that regulatory elements were induced by admixture.

### The admixture-induced risk of immune-related diseases

We constructed an admixture-induced gene expression model to illustrate the expression modes and their regulatory effects on the admixed group (Fig. 5B). The model was simplified as a regulator-expression model: before admixture, the genes were expressed differently in each ancestry, while after long-term admixture, the gene expression levels were in an intermediate state in the admixed group when compared with the two ancestral populations, which were defined as the ideal state. Otherwise, the state was defined as an admixed group-specific state. In addition, the state differences could be due to the joint regulatory effects of genetics and ancestral background. This model could also be applied to these joint regulatory effects: before admixture, the regulatory effects of the ancestries were different; and after long-term admixture, these regulatory effects could be in the intermediate state or the admixed group-specific state (see Materials and Methods and Supplementay Note S3).

We applied this model to XJU using the ancestral source groups EUR and EAS to examine the admixed-induced genetic effects on gene expression. At the whole genome level, 5 347 178 SNP-gene pairs were tested via this model. Only ∼0.12% significantly fit the model. Among these fitted SNP-gene pairs, the majority (∼62%) were identified in the XJU-specific state (∼32% were in an enhanced state, and ∼30% were in a relaxed state), and only ∼38% were in an ideal state. Approximately ∼10% of SNPs that fitted our model were highly differentiated east–west variants. Furthermore, 50% of the associated genes were in an ideal state. The diseases related to genes in an ideal state and those in other specific states were highly similar (Supplementay Figs S40 and S41). Most of the genes were related to immune and metabolic diseases; these diseases were covered in our above observations, for example, T1D, leishmaniasis, IBD, AITD and TB. The results imply that the genetic admixture and adaptation coaffected the phenotypes of XJU.

The most notable candidate enhancement regulator was rs11257655, the lead-eQTL for *NUDT5* in XJU but not in EAS or EUR [and that could not be found in the GTEx database for whole blood [53]] located 70 kb upstream of the gene in the enhancer region [54]. This eQTL showed high ancestral genetic differentiation (${F}_{ST}$ between EAS and EUR = 0.18). The rs11257655*-T* allele is a risk allele related to T2D in the European [55,56] and Asian [57,58] populations. *NUDT5* was involved in the metabolism and metabolism of nucleotides pathways. Our findings indicate that the admixture increases the genetic diversity of this variant, thus imposing the genetic regulation of *NUDT5* at the expression level (Fig. 5C), making this SNP more adaptive in XJU.

Other examples, such as rs7115703, highlighted the influence of western ancestry on gene expression and possible phenotypic variation in the admixed population. The *IFITM3* gene encodes an interferon-induced membrane protein that facilitates immunity to influenza, an H1N1 virus, the West Nile virus and the dengue virus. The related pathways were associated with the innate immune system. There were 56 *cis*-eQTLs of *IFITM3*, of which 32 showed a strong signature of ancestral genetic divergence (${F}_{ST}$ between EAS and EUR > 0.4, Supplementay Fig. S42). The lead eQTL, (rs7115703 *T*/*A*), located 13 kb upstream of this gene, exhibited an extreme discrepancy (*T* allele frequency of EUR and EAS: 0.44 and 0.009, respectively): the *T* allele was nearly fixed in EAS, suggesting that this *T* allele was derived from western ancestors. *IFITM3* was downregulated by rs7115703*-T*, similar to that in the EUR, but to a higher degree (Supplementay Fig. S43). The rs7115703 is an eQTL in many tissues in EUR [59] that was reported to be more likely to be causally [60] related to the granulocyte percentage of myeloid white cells in one GWAS [61].

One example of a candidate relaxation regulator gene was *PEX6* on chromosome 6 encoding a member of the AAA [adenosine triphosphatases (ATPases) associated with diverse cellular activities] family of ATPases [62]. This gene was associated with 53 highly linked candidate relaxation regulators (${r}^2{\rm{\ }} > {\rm{\ }}0.8$) that were eQTLs in both EAS and EUR and were HDS-eQTLs in XJU (Fig. 5D). The ES of these HDS-eQTLs was weaker in XJU than in EAS or EUR (*P* < 0.05; Fig. 5E). Although these HDS-eQTLs were not risk variants of *PEX6* [63], our results suggest that the heterogeneity of the *PEX6* gene between XJU and its ancestral source populations might be related to genetics.

## DISCUSSION

Our study focused on the regulatory effects of the highly differentiated variants and genes between the east and west, thereby providing a comprehensive understanding of the genetic regulation of eastern and western genes and also illustrating the influence of genetic admixture and population adaptations on transcriptomes. We identified hundreds of highly differentiated regulatory variants and genes with their potential regulatory effects on expression and phenotypes. Our analysis suggested that many of the highly differentiated variants played regulatory roles by altering gene expression and causing alternative splicing in genes associated with several traits and diseases. We detected unbalanced allele expression and found that the western ancestral background of the alleles was a possible factor associated with allele overexpression. The genes significantly associated with these loci were tightly connected in functional networks and immune- or metabolism-related diseases, as previously reported in XJU, including T1D, NAFLD, TB, IBD, asthma, AITD and leishmaniasis. We speculated that the prevalence of these diseases in XJU was influenced by the pleiotropic effects of the risk loci or genes that were highly differentiated in Eastern and Western populations, possibly underlying natural selection, and with relatively large regulatory effects. Genetic admixture plays an important role in gene expression regulation by combining risk alleles of different ethnic origins and increasing disease risk prevalence. In addition to admixture effects, adaptation to local environments is another risk factor, considering the dietary habits of XJU. High-fat and high-energy habits, the consumption of dairy products, and red-meat consumption facilitate a distinct gut microbiome of XJU [64] that could be correlated with host genetic variants, and thus affecting numerous pathological conditions, such as ulcerative colitis, Crohn's disease, IBD, colorectal cancer, obesity, diabetes and nonalcoholic steatohepatitis [65].

We further explored the admixture-induced expression regulatory effects in the admixed group. When individuals were grouped according to ancestry proportions at each gene, 825 genes showed a significant correlation between expression patterns and ancestry. There was a tendency that the expression levels of the admixed group were between that of the eastern ancestry-like group and that of the western ancestry-like group (one-tailed *t* test, *P* < 0.05). On the basis of those observations, an admixture-induced gene expression model was proposed (Fig. 5B). To understand the mechanisms of the expression patterns, we modeled the joint effects of the variants and their ancestry on gene expression level, furthering our knowledge of the pleiotropic effects of highly differentiated alleles on molecular etiology and providing new insights into genetic differences associated with substantially differentiated phenotypes between Eastern and Western populations. We anticipate that analyses of gene expression profiles and their genetic regulators in the Uyghurs advance our understanding of population admixture as a driving force in human phenotypic evolution.

## MATERIALS AND METHODS 

### Samples information and processing

The study was approved by the Biomedical Research Ethics Committee of Shanghai Institutes for Biological Sciences (No. ER-SIBS-261408), and the samples applied in this study, 90 XJU individuals and 40 Han Chinese (hereafter HAN), were collected with informed consent. The personal identifiers of all samples, if any existed, were stripped before sequencing and analysis. All procedures followed the ethical standards of the Responsible Committee on Human Experimentation and the Helsinki Declaration of 1975, as revised in 2000.

The RNA sequencing data of samples was extracted using a PAXgene Blood RNA Kit (QIAGEN) and sequenced on the Illumina HiSeq2000 platform. On average, each individual could be identified with 12 million 100-base pair (bp) unstrand paired-end reads, and the coverage ratio was ∼30× for the exon region across the genome (Table S1). The whole genome sequencing was performed with the Illumina Hi-Seq platform. The detailed sample information, data processing and quality control was described in the supplementary method part and our previous study [45].

### Ancestral source population identification

For simplification, we regarded XJU as having two major ancestry components, i.e. the West and the East Eurasian, as identified in previous studies [7,30]. We used the Han Chinese in Beijing population (CHB) and the British population in England and Scotland (GBR), two typical East and West Eurasian populations, as ancestral source populations of XJU. The CHB and GBR population samples were collected from the GEUVADIS project [3,4,12]. CHB and GBR were referred to as EAS and EUR, respectively, in this study.

### HDS and HDG estimation

We measured the genetic difference at the population level using ${F}_{ST}$ following Weir and Cockerham [66]. At the site level, the SNPs with ${F}_{ST} > 0.2$ were defined as highly differentiated SNPs and labeled as HDS. Other SNPs at the whole-genome level were defined as non-HDS. Each gene with both a mean$\ {F}_{ST} > 0.2,$ and a weighted ${F}_{ST} > 0.2$, were defined as highly differentiated and labeled as HDG.

### Modeling admixture-induced effects

We considered an admixture-induced gene expression model to illustrate the expression modes and their regulatory effects of the two-way admixed group as two parts:

1. Regarding the gene expression, the ancestries were expressed differently at the population level. Expression level in the admixed group between that of its ancestral populations after long-term admixture was defined as the ideal state. Otherwise, the expression level was defined as under the admixed group-specific state.
2. Regarding the expression regulation, we hypothesized that genetics and ancestral backgrounds jointly affected gene expressions.

We analyzed the covariance between XJU and its ancestral source populations to test whether the change in ES was in the ideal state. When *P* < 0.05, the ES changes were not in the ideal state, and we further compared the degree of change (beta value) in the R package ‘MatrixEQTL’ [67] to determine the direction of the change in ES. Forty individuals were randomly sampled from each group to guarantee a comparable sample size.

Detailed descriptions of the methods are available in the Supplementary Data.

## DATA AND MATERIALS AVAILABILITY

All data needed to evaluate the conclusions in the paper are present in the paper and the Supplementary Materials. The eQTLs summary statistics of XJU and HAN can be accessed at NODE (http://www.biosino.org/node) with the accession number OER266226 (XJU) and OER295224 (HAN), and accessed at NGDC (https://ngdc.cncb.ac.cn/omix/) with the accession number OMIX002320. The use of the data by this work is approved by the Ministry of Science and Technology of the People's Republic of China (2022BAT2507 and 2022BAT1947). The computer code of the workflow for expression files quantification and comparison can be found on the group website (https://pog.fudan.edu.cn/#/software), GitHub repository (https://github.com/Shuhua-Group/RNA-Seq) and Zenodo (https://doi.org/10.5281/zenodo.7313079).

## Supplementary Material

nwad077_Supplemental_File
