# Supplementary material for: Expression profiles of east–west highly differentiated genes in Uyghur genomes
Source: Natl Sci Rev. 2023 Mar 21;10(4):nwad077. doi: 10.1093/nsr/nwad077 (PMC10150800; doi:10.1093/nsr/nwad077)
Supplement: nwad077_Supplemental_File [file nwad077_supplemental_file.docx]

Supplementary Materials

**Expression profiles of east-west highly differentiated genes in Uyghur genomes**

*Teaser: East-west differentiated gene expression profiles*

*Zhilin Ning^1,^* †*, Xinjiang Tan^1,^* †*, Yuan Yuan^1,^* †*, Ke Huang^1,2^, Yuwen Pan^1^, Lei Tian^1^, Yan Lu^3^, Xiaoji Wang^1^, Ruicheng Qi^1^, Dongsheng Lu^1^, Yajun Yang^3^, Yaqun Guan^5^, Dolikun Mamatyusupu^6^, Shuhua Xu^3,4,7^**

^1^Key Laboratory of Computational Biology, Shanghai Institute of Nutrition and Health, University of Chinese Academy of Sciences, Chinese Academy of Sciences, Shanghai 200031, China;

^2^School of Life Science and Technology, Shanghai Tech University, Shanghai 201210, China;

^3^State Key Laboratory of Genetic Engineering, Center for Evolutionary Biology, Collaborative Innovation Center of Genetics and Development, School of Life Sciences, Fudan University, Shanghai 200438, China;

^4^Human Phenome Institute, Zhangjiang Fudan International Innovation Center, and Ministry of Education Key Laboratory of Contemporary Anthropology, Fudan University, Shanghai 201203, China;

^5^Department of Biochemistry and Molecular Biology, Preclinical Medicine College, Xinjiang Medical University, Urumqi 830011, China;

^6^College of the Life Sciences and Technology, Xinjiang University, Urumqi 830046, China;

^7^Department of Liver Surgery and Transplantation, Liver Cancer Institute, Zhongshan Hospital, Fudan University, Shanghai 200032, China.

†These authors contributed equally to this work.

*Correspondence and requests for materials should be addressed to S.X. (Email: [xushua@fudan.edu.cn](mailto:xushua@fudan.edu.cn)).

Contents

[*Teaser: East-west differentiated gene expression profiles* 1](#_Toc128556867)

[Supplementary Notes 4](#_Toc128556868)

[Note S1. The information on the in-house Han Chinese population. 5](#_Toc128556869)

[Note S2. The batch effects detection among different populations. 6](#_Toc128556870)

[Note S3. The admixture-induced gene expression model of the two-way admixed group. 7](#_Toc128556871)

[Note S4. Detailed methods 8](#_Toc128556872)

[Supplementary Figures 17](#_Toc128556873)

[Fig. S1 | The distribution of successful mapping rates of XJU. 18](#_Toc128556874)

[Fig. S2 | Principal component analysis (PCA) for XJU, east and west Eurasian in DNA level. 19](#_Toc128556875)

[Fig. S3 | The PCA plot of gene expression. 20](#_Toc128556876)

[Fig. S4 | PCA for XJU, east and west Eurasian, and other populations in gene expression level. 21](#_Toc128556877)

[Fig S5 | PCA for XJU, east and west Eurasian, and other populations in AS events level. 22](#_Toc128556878)

[Fig S6 | PCA for XJU, east and west Eurasian, and other populations in AS data after the subgroup was removed. 23](#_Toc128556879)

[Fig S7 | PCA for XJU, east and west Eurasian, and other populations in transcriptome level with AS data after the subgroup was removed and PEER normalization. 24](#_Toc128556880)

[Fig S8 | Diagnostic plot of the hidden factor relevance (ARD parameters) in PEER 25](#_Toc128556881)

[Fig S9 | The functional elements enrichment of HDSs in XJU. 26](#_Toc128556882)

[Fig S10 | The GWAS enrichment of HDSs in XJU. 27](#_Toc128556883)

[Fig S11 | The GWAS enrichment of HDSs-covered genes in XJU. 28](#_Toc128556884)

[Fig S12 | The KEGG enrichment of HDSs-covered genes in XJU. 29](#_Toc128556885)

[Fig S13 | The distribution of HDGs in XJU. 30](#_Toc128556886)

[The HDGs were exhibited in each chromosome by Rishishwar, Conley [41]. 30](#_Toc128556887)

[Fig S14 | The GWAS enrichment of HDGs in XJU. 31](#_Toc128556888)

[Fig S15 | The CADD comparison between HDSs and non-HDSs in XJU. A Student *t* test was conducted to compare the differences. 32](#_Toc128556889)

[Fig S16 | The comparison of ancestral genetic differences between eQTLs and non-eQTLs 33](#_Toc128556890)

[Fig S17 | The distribution of ancestral genetic difference vs. QTLs percentages in XJU. 34](#_Toc128556891)

[Fig S18 | The distribution of ancestral genetic difference vs. QTLs percentages in XJU. 35](#_Toc128556892)

[Fig S19 | The functional elements enrichment of HDS-eQTLs in XJU. 36](#_Toc128556893)

[Fig S20 | The functional elements enrichment of HDS-sQTLs in XJU. 37](#_Toc128556894)

[Fig S21 | The GWAS enrichment of HDS-eQTLs in XJU. 38](#_Toc128556895)

[Fig S22 | The GWAS enrichment of HDS-sQTLs in XJU. 39](#_Toc128556896)

[Fig S23 | The functional elements enrichment of HDS-ASE in XJU. 40](#_Toc128556897)

[Fig S24 | The GWAS enrichment of HDS-ASE in XJU. 41](#_Toc128556898)

[Fig S25 | The KEGG enrichment of HDS-aseGenes in XJU. 42](#_Toc128556899)

[Fig S26 | The GWAS enrichment of HDS-aseGenes in XJU. 43](#_Toc128556900)

[Fig S27 | The distribution of ancestral genetic difference vs. ASE percentages in XJU. 44](#_Toc128556901)

[Fig S28 | The comparison of ancestral genetic differences between ASE and non-ASE with *t* test. 45](#_Toc128556902)

[Fig S29 | The comparison of the degree of allelic imbalanced expression (AI) among all ASE. 46](#_Toc128556903)

[Fig S30 | The functional elements enrichment of HDS-aseQTLs in XJU. 47](#_Toc128556904)

[Fig S31 | HDSs with a higher degree of deviation indicated evidence of adaptation. 48](#_Toc128556905)

[Fig S32 | The density plots of CADD and GERP scores of HDSs conditioned on the degree of the deviation. 49](#_Toc128556906)

[Fig S33 | HDSs with putative regulatory functions (HDS-QTLs and HDS-ASE) showed a higher degree of deviation compared with HDSs without putative regulatory functions. 50](#_Toc128556907)

[Fig S34 | The functional elements enrichment of the HDSs with multiple putative regulatory functions and possibly having undergone harder selective pressure in XJU. 51](#_Toc128556908)

[Fig S35 | The distribution of effect size of all eQTLs in XJU. 52](#_Toc128556909)

[Fig S36 | The KEGG pathway network of HDSE-gene in XJU. 53](#_Toc128556910)

[Fig S37 | The schematic diagram of aseQTLs of two ASEs on *UTS2.* 54](#_Toc128556911)

[Fig S38 | The associated ancestral inferred segments of XJU. 56](#_Toc128556912)

[Fig S39 | The associated ancestral inferred segments of XJU. 58](#_Toc128556913)

[Fig S40 | The KEGG enrichment of the associated genes modeled in the ideal state. 59](#_Toc128556914)

[Fig S41 | The KEGG enrichment of the associated genes modeled in the specific state (relaxed or enhanced). 60](#_Toc128556915)

[Fig S42 | The 56 eQTLs of *IFITM3* in XJU. 61](#_Toc128556916)

[Fig S43 | The associations between rs711570 and *IFITM3* in XJU, EAS, and EUR. 62](#_Toc128556917)

[The x-axis indicated the genotypes. 62](#_Toc128556918)

[Supplementary Tables 63](#_Toc128556919)

[Table S1 | The sequencing properties of XJU and HAN per individual. 64](#_Toc128556920)

[Table S2 | The sample size information of applied populations in the 1KG dataset. 68](#_Toc128556921)

[Table S3 | The properties of ASE detection at XJU individual level. 69](#_Toc128556922)

[Table S4 | The 31 ancestral-like genes were eQTLAS-genes in XJU. 72](#_Toc128556923)

[Table S5 | The 144 collected T2D-related studies applied in our T2D enrichment analysis. 72](#_Toc128556924)

[Reference 73](#_Toc128556925)

Supplementary Notes

Note S1. The information on the in-house Han Chinese population.

The RNA of 40 Han Chinese individuals (hereafter labeled as HAN) was generated together with XJU, which was extracted using a PAXgene Blood RNA Kit (QIAGEN) and sequenced on the Illumina HiSeq2000 platform. The sequencing data were processed by the same pipeline described in the method part to quantify the expression data and detect eQTLs. And the HAN SNVs were extracted from the sequencing dataset curated by our lab (see methods). In total, there were 7,174,375 SNPs in HAN. This population was applied as a reference to check the batch effects between XJU and populations from the E-GEUV-1-RNA-sequencing dataset, and to detect the eQTLs. The eQTLs association was processed as described in the method part. The effect size of the eQTLs were referred as the joint regulatory effect of the eastern ancestry to be applied in the admixture-induced expression model of XJU. And we check the effect size of the SNPs, which were detected as eQTLs in XJU, in HAN by the results of the eQTLs,

Note S2. The batch effects detection among different populations.

Batch effects were checked among 7 populations, including XJU, HAN, and populations from the E-GEUV-1-RNA-sequencing dataset (including European-ancestral populations GBR, CEU, TSI, and FIN as well as African-ancestral population YRI) in genome and transcriptome level. At the genome level, PCA was performed to validate the population relationship (Fig. S2) with smartpca [1, 2] using 4,772,642 autosome biallelic SNPs (MAF > 0.01) identified in 7 populations. The pattern was under expectation based on previous analysis in our lab [3, 4], which indicated that there was no batch effect between XJU and populations in the E-GEUV-1-RNA-sequencing dataset, and the quality of the genotype data of XJU and HAN was relatively high.

At the transcriptome level, gene expression data and AS events data were applied to check the batch effects among 7 populations. Only autosome genes were considered. First, For gene expression data, in total, there were 21,008 autosome genes applied. The potential batch effect existed between different datasets (dataset generated by our lab and E-GEUV-1-RNA-sequencing dataset, Fig. S3). And PEER failed to remove the batch effect. While the potential batch effect within each dataset could be removed relatively perfectly (Fig. S4). Then, for AS events data, autosome AS events was considered. PCA was conducted in each type of AS (SE, IR, A5SS, A3SS), including 47,873 SE events, 2,079 IR events, 2,106 A5SS events, and 3,027 A3SS events. We observed the potential batch effects between the two datasets, and also within the E-GEUV-1-RNA-sequencing dataset (Fig. S5). We found that samples generated in lab 2 from the E-GEUV-1-RNA-sequencing dataset clustered together, which were considered outliers and removed in our downstream analysis. PEER relatively succeed to remove the potential batch effects within the E-GEUV-1-RNA-sequencing dataset but failed to remove the potential batch effects between datasets (Fig. S6-7). So the irremovable batch effects observed in transcriptome level made us decide to conduct downstream analysis within the population instead of across populations, and also alert us to interpret the comparative results carefully.

Note S3. The admixture-induced gene expression model of the two-way admixed group.

The admixture-induced gene expression model was constructed to illustrate the expression modes and dissect the regulatory effects from the ancestries to the admixed group (Fig. 5B). The model was simplified as a regulator-expression model, including two parts. In the first part, we used the model to describe the admixture-induced expression modes from the ancestries to the admixed group: before admixture, the ancestries were expressed differently; after long-term admixture, the gene expressions were in an intermediate state compared with the two ancestral populations. We defined this state as the ideal state. Otherwise, the state was defined as an admixed group-specific state. In the second part, we tried to dissect the potential mechanisms of the fluctuations of expressions from ancestral to the admixed group, we considered$S_{0}$ as a single SNP inherited from ancestors to the admixed group. The expression level of$S_{0}$ affected genes were jointly influenced by$S_{0}$ with its ancestry. To simplify, we used$S_{0}$ to label this joint effect between the SNP and its ancestry. We expected the joint regulatory effect of$S_{0}$ in the admixed group was between those of the two ancestors, which made the gene expression level in the ideal state. Otherwise, the gene expression level was affected in an admixed group-specific state: (1) when the joint effect of$S_{0}$ was larger than expected, the state was referred to as the admixed group enhanced state, and$S_{0}$ was defined as the regulation enhancement regulatory label; (2)otherwise, the state was referred to as the admixed group relaxed state,$S_{0}$ was defined as the regulation relaxation regulatory label. Using this model, we could integrate the effects of genetics and ancestry to detect candidate adaptation signals of the admixed group at the population transcriptomic level (Methods).

Note S4. Detailed methods

**RNA-sequencing and data processing**

The RNA of 90 XJU individuals and 40 Han Chinese individuals (hereafter HAN) was extracted using a PAXgene Blood RNA Kit (QIAGEN) and sequenced on the Illumina HiSeq2000 platform. On average, each individual could be identified with 12 million 100-base pair (bp) unstrand paired-end reads, and the coverage ratio was ~30× for the exon region across the genome (Table S1), which was estimated as:

$Coverage Ratio per Individual = \frac{N_{mapped}\times L_{mapped}}{2 \times L_{exon}}$ (1)

where$N_{mapped}$indicates the number of uniquely mapped reads,$L_{mapped}$ indicates the average mapped length, an$L_{exon}$ indicates the total length of exon regions (34,729,283 bp) across the genome, which was estimated from the Consensus Coding Sequence (CCDS) [5] of National Center for Biotechnology Information (NCBI). Parameter *2* was applied because the reads were generated by pair-end sequencing. The reads were mapped to the human reference genome (Hg19) using STAR [6]. The successful mapping rate was ~92.43% (minimum 83.78%; maximum 93.74%, Supplementay Figure 1). Next, RSEM [7] was used to quantify and normalize gene and transcript expression. We used FPKM (fragments per kilobase of transcript per million mapped reads) to quantify expression levels. One gene or transcript was regarded to be expressed when at least 20 samples showed FPKM > 0.01 within each population. Alternative splicing (AS) events were detected with rMATs [8]. One event with AS was defined when the percent spliced-in (PSI) value ranged from 0.1 to 0.9. In total, in XJU, there were 22,794 genes and 1,547 AS events applied to the downstream QTL analysis; in HAN, there were 218,690 genes applied to the downstream eQTL analysis.

All samples were collected with informed consent and the study was approved by the Biomedical Research Ethics Committee of Shanghai Institutes for Biological Sciences (No. ER-SIBS-261408). The personal identifiers of all samples, if any existed, were stripped before sequencing and analysis. All procedures were followed the ethical standards of the Responsible Committee on Human Experimentation and the Helsinki Declaration of 1975, as revised in 2000.

**Genome sequencing and data processing**

Genome sequencing was performed with the Illumina Hi-Seq platform for the 90 XJU samples; detailed sample information is described in our previous study [3]. The genotype imputation of XJU and KGP was conducted with IMPUTE2 (version: 2.3.2) [9] to avoid potential batch effects and fully reflect the XJU admixture characteristics. The output files were transformed into a variant call format (VCF) format by PLINK v1.9 [10] and qctool (version: v1.4-Linux-x86_64; <https://www.well.ox.ac.uk/~gav/qctool>). Two reference panels were used: one from the dataset of 1000 Genomes Project Phase III dataset, including 77,818,183 sites and 2,504 individuals, and one from a sequencing dataset curated by our laboratory, including Han Chinese [11], Uyghurs[3], Tibetans [12, 13], Huis [14], and other East Asian populations [15], including 29,394,607 sites and 1,025 individuals. After imputation, 32,025,769 sites and 14,262,138 SNPs in XJU were retained.

**Data quality control**

After the above data pre-processing, we obtained genotype data and quantified expression data. Then, we verified populations and estimated sample quality based on principal components analysis (PCA). Population and/or sample outliers that were in conflict with self-reporting and existing batch effects can be excluded. PCA was performed at genomic and transcriptome levels with genotype, expression, and AS events data (Supplementay Note S2 and Supplementay Figures 2 to S7) by smartpca [2, 16] and the R function “procomp” in the “base” package. No samples were excluded from our research. The sample size information applied in our research was shown in Table S2.

**Transcriptome QTL mapping**

Transcriptome QTLs were associated with each population. For genotype data, heterozygotes were retained when the missing rate was < 10% and the Hardy-Weinberg disequilibrium was < 10^−16^ by PLINK. Rare SNPs were defined as minor allele frequency (MAF) < 1%, and common SNPs were defined as MAF > 1%. Expressed genes were retained (see above Materials and Methods). There were 14,255,892 SNPs after the quality control. Gene expression was quantified after PEER [17] correction to improve detection power, in which sex and age were set as common covariates, and hidden factors were set at three (Supplementay Figure 8).

To detect eQTLs, we performed an association analysis between adjusted expression values of genes and SNPs with a linear regression model in the R package “MatrixEQTL” [18] using sex and age as common covariates. We only considered *cis* association here: SNPs within 100 kb of gene boundaries on the same chromosome. The 100-kb gene boundary extensive size was considered for the detecting of the *cis* eQTLs with strong effects and reducing of the burden of multiple testing. An SNP was defined as an eQTL when ES > 0.2 and adjusted *P* < 0.05. A gene was defined as an eGene when it was associated with one or more eQTLs.

**Ancestral source populations**

As previously reported, XJU was modeled as an admixed population of four major ancestral components potentially derived from two earlier admixed groups: one from the West, including European and South Asian ancestries, and the other from the East with Siberian and East Asian ancestries [4]. For simplification, we regarded XJU as having two major ancestry components, i.e., the West and the East Eurasian, as identified in previous studies [19, 20]. We used the Han Chinese in Beijing population (CHB) and the British population in England and Scotland (GBR), two typical East and West Eurasian populations, as ancestral source populations of XJU. The CHB and GBR population samples were collected from the GEUVADIS project [21-23]. CHB and GBR were referred to as EAS and EUR, respectively, in this study.

We collected genotype data of GBR and CHB from the 1000 Genomes Project Phase III dataset. Only SNPs detected in XJU, CHB, and GBR were applied in the HDSs analysis. The expression data of GBR was generated from fastq files of lymphoblastoid cell lines, which were downloaded from the E-GEUV-1-RNA-sequencing dataset [22]. We processed the initial sequences in the same way as XJU. There were ~30Mb reads for a pair-end sequenced sample, of which ~33% had adapters, and ~2% of the sequence nucleotides were trimmed. The successful mapping rate was ~87.76% (minimum 55.31%; maximum 91.28%). GBR and other EUR populations [including the Utah Residents (CEU), Finnish (FIN), Tuscan (TSI), and Yoruba (YRI)] in the KGP dataset were processed in the same way.

**Estimation of HDS and HDG**

We measured the genetic difference at the population level using$F_{ST}$ following [Weir and Cockerham (1984)](javascript:;), a statistic that accounts for differences in sample sizes between populations. At the site level, we calculated$F_{ST}$ between ancestral source populations to determine genetic differences in an admixed population. The average$F_{ST}$ of SNPs is 0.0091 (SD = 0.04) across genomes. We used${mean(F}_{ST})+ 5 \times sd{(F}_{ST})$ as the threshold to define HDS, which approximately equals 0.2. In addition,variants with$F_{ST}>0.2$ account for the top ~1% out of all variants. The SNPs with$F_{ST}>0.2$ were defined as highly differentiated SNPs and labeled as HDS. Others SNPs at the whole-genome level were defined as non-HDS. For each gene, to estimate the value of mean and weighted$F_{ST}$ for each gene, SNPs located on each gene containing all the variants between the two UTRs, were selected as an SNP block. We calculated the mean$F_{ST}$ and the weighted$F_{ST}$ using VCFtools [24]. The average$F_{ST}$ of the genes was 0.06 (SD = 0.06). For the consistency of thresholds, genes with both a mean$F_{ST}>0.2,$ and a weighted$F_{ST}>0.2$, were defined as highly differentiated and labeled as HDG.

**Estimation of allele frequency deviations**

We measured the allele frequency deviation of the SNPs between the observed allele frequency and the expected allele frequency of SNPs as

$$\Delta AF = \left| {AF}_{expected}-{AF}_{observed} \right| (2)$$

where${AF}_{observed}$ was the observed allele frequency in XJU and${AF}_{expected}$ was calculated as${AF}_{expected}=f_{East}\times a+f_{West}\times(1-a)$. Here, $a$ is the overall admixture proportion of ancestry A in XJU, which was 0.5 as reported previously [19, 20]; $f_{East}$ is the allele frequency in the eastern source ancestral population of XJU, and$f_{West}$ is the allele frequency in the western source ancestral population of XJU.

**Functional annotation and enrichment analysis of regulated variants and genes**

Functional enrichment analysis of associated variants (including eQTLs, sQTLs, and aseQTLs) was performed using Fisher’s Exact Test with Ensembl Regulatory Build and coding annotations [25]. We tested for the enrichment of these associated variants relative to all the variants in the *cis* region of genes on the genome as a null set. The degree of enrichment was measured by the odds ratio (OR). Relevant variants were identified as enriched in the functional category if the OR was > 1 and the adjusted *P* value was < 0.05 on Fisher's exact test.

Positional enrichment of associated variants was performed on different chromosomes. We used a 1 Mbp wide sliding window using a step size of 100 kbp across each chromosome to calculate the number of regulatory variants and presented the results via Manhattan plots. Predicted function enrichment of associated genes (including eGenes and sGenes) was performed with the R packages “clusterprofiler” [26] and “msigdbr ” [27-29] (C2 curated gene sets from MSigDB).

To estimate the traits or diseases related to genes, we conducted an enrichment analysis between interested genes and GWAS traits collected from the GWAS catalog [30] as introduced in the previous study [31]. Because GWAS have different distributions of data profiles and *P* values, we used a set of cutoffs ranging from${10}^{-30}$ to${10}^{-8}$. For each GWAS trait, Fisher’s exact test was applied to calculate the *P* value and to estimate the enrichment degree.

For the enrichment analysis of T2D, we used 1,138 T2D-susceptible genes and 3,120 loci with T2D-susceptible alleles from 144 studies (Table S5), T2D-susceptible genes and variants reported in GWAS Catalog [32] and other studies and the T2D specific research [33] as the repertoire of T2D-susceptible genes and variants. Next, we used Fisher’s exact test to test the enrichment results.

**ASE quantification and aseQTLs identification**

ASE analysis was based on a binomial test of each allelic ratio of heterozygous sites per individual within each population. RNA-seq reads were aligned to hg19 with allelic mapping bias correction using STAR 2.7.3a [6] in two-pass mode. SNP-level ASE data were generated using phASER v3.22.0 [34]. To increase the power of ASE calling, we only considered heterozygous sites with at least 10 reads to avoid noise caused by insufficient coverage. The ratio of AI for each site was defined as the reads proportion of minor alleles among all reads:

$\left| \frac{ref}{ref+alt} - 0.5 \right| (3)$

where $ref$ and $alt$ are the numbers of mappable reads of reference and alternative alleles. One ASE locus (aseSNPs) was defined as false discovery rate (FDR) < 5% in the binomial test and AI ≥ 0.2.

Differential expression of the two haplotypes of an individual, or allele-specific imbalanced expression, was possibly driven by two major factors: epigenetics and genetics. The genetic-driven factors were considered *cis* genetic regulators and nonsense SNPs in which protein-truncating variants caused nonsense-mediated decay (NMD) on the ASE loci [34, 35]. When an ASE event was driven by *cis* genetic regulators, regulatory SNPs in the *cis* region of ASE loci were defined as regulatory SNPs (rSNPs) [36]. At the population level, we defined aseQTLs when individuals heterozygotes for that candidate aseQTLs in the *cis* region (100 kb flanking the ASE loci) showed a higher ratio of AI than homozygotes for identified ASE loci in the one-sided Mann–Whitney *U* test. The ASE loci with total reads counts ≥ 10 in at least 10 individuals and heterozygous SNPs within its *cis* region were retained in the associations.. The aseQTLs were defined as those with a locally adjusted *P* value < 0.05.

**Local ancestry inference and admixture mapping**

The local ancestry inference of XJU was conducted with Loter [37], a program suitable for data with an extremely high variant density and multi-way admixture scenarios. The merged data set was prepared for local ancestry inference with default parameters, including whole-genome sequencing data of XJU and reference populations. Ninety-nine individuals randomly sampled from both CHB and CEU in the 1000 Genomes Project Phase III dataset for the two-way admixture model, as in our previous research [4, 19, 38], were used as reference data representing the ancestral Eastern and Western populations. We inferred the probability of the eastern ancestral copy number (0, 1, or 2) at each location in the genomes of XJU individuals. The weighted sum of these probabilities (multiplied by 0, 0.5, or 1) formed an estimate of the local ancestral proportion. Genome-wide ancestry was computed as the average estimated local ancestry proportion throughout the genome. Adjacent SNPs were merged into one specific ancestry segment with consistent ancestry proportions. There were 214,652 merged segments in total.

Next, the local ancestral proportions of the remaining segments were imputed based on the adjacent segments. In each individual, if the adjacent segments were derived from the same ancestor, then the segments and the gap between them could be merged as one segment. If the adjacent segments were derived from different ancestors, then the gap would be divided evenly into two segments and merged into the closest segment separately. For each chromosome, the upper boundary of the first segment and the lower boundary of the last segment were extended to the start and the end positions of the chromosomes. Therefore, we could obtain the local ancestral proportion of the whole genome in each individual. Then, we calculated the local ancestral proportion of each gene as

$$P_{Eastern} = \frac{L_{Eastern}}{L_{Eastern}+L_{Western}} (4)$$

where$L_{Eastern}$ is the length of the segments derived from eastern ancestry and$L_{Western}$ is the length of the segments derived from western ancestry. $P_{Eastern}$ was defined as the eastern ancestry proportion of genes. $P_{Westerm}$ was defined as the western ancestry proportion of genes.

For each gene, the individuals could be divided into three groups based on the gene ancestry proportions: (i) an eastern ancestry-like group, individuals with$P_{Eastern}> 0.7$; (ii) a western ancestry-like group, individuals with$P_{Eastern}$ < 0.3; and (iii) the admixed group, individuals with 0.3 $\leq P_{Eastern}\leq0.7$. We conducted analysis of variance (ANOVA) to dissect the differences in gene expression levels among the three groups per gene. In addition, we used a one-tailed *t* test to compare the expression levels between pairwise groups, including the expression level between the eastern ancestry-like and the admixed populations, the western ancestry-like and the admixed populations, the admixed group, and the other two groups. The eastern ancestry-like genes were defined as genes with expression levels that were significantly higher in the eastern ancestry-like group than in the other two groups (ANOVA adjusted *P* < 0.05; *t* test *P* < 0.05). The western ancestry-like genes were defined as genes with expression levels significantly higher in the western ancestry-like group than in the other two groups (ANOVA adjusted *P* < 0.05; *t* test *P* < 0.05).

We further associated the ancestry proportions of these merged segments with expressed genes with “MatrixEQTL” [18] similar to the above methods for QTL detection. Additionally, we only considered *cis* association here: segment boundaries within 100 kb from gene boundaries on the same chromosome. A segment was defined as an eQTAS when the adjusted *P* value was < 0.05. A gene was defined as an eQTAS-gene when it was associated with at least one more eQTAS.

**Modeling admixture-induced effects**

On the basis of our observations, we considered an admixture-induced gene expression model to illustrate the expression modes and their regulatory effects of the two-way admixed group. We considered the model in two parts:

1) Regarding the gene expression, the ancestries were expressed differently at the population level. Expression level in the admixed group between that of its ancestral populations after long-term admixture was defined as the ideal state. Otherwise, the expression level was defined as under the admixed group-specific state.

$$exp state\left( admixed \right) = \left\{ \begin{aligned} exp\left( Anc1 \right) < exp\left( admixed \right) < exp(Anc2), the ideal state \\ exp\left( admixed \right) >max exp\left( Anc1, Anc2 \right) or exp\left( admixed \right) < min exp\left( Anc1, Anc2 \right), the admixed group-specific state \end{aligned} \right. (5)$$

(2) Regarding the expression regulation, we hypothesized that genetics and ancestral backgrounds jointly affected gene expressions. To simplify the complex admixture events and the joint effects across the genome, we considered a single SNP, $S_{0}$, inherited from ancestors to be the admixed group, and then $S_{0}$-affected genes were jointly influenced by $S_{0}$ with its genetic background. Here, we used $S_{0}$ to label this joint effect to model the potential mechanisms of the expression changes from the ancestral groups to the admixed group. When the joint effects of$S_{0}$ in the admixed group was between those of the two ancestral populations, gene expression levels were considered to be in the ideal state. Otherwise, the joint effects of$S_{0}$ considered to be in an admixed group-specific state. Furthermore, in the admixed group-specific state, when the joint effect of$S_{0}$ was larger than expected, the state was defined as the admixed group enhanced state, and$S_{0}$ was defined as the regulation enhancement regulatory label; otherwise, the state was defined as the admixed group relaxed state, and $S_{0}$ was defined as the regulation relaxation regulatory label. The expected joint effect of $S_{0}$ in the admixed group was defined as:

$${expected joint effect \mathrm{of}S_{0}}_{admixed} = m\times{joint effect \mathrm{of}S_{0}}_{Anc1}+\left( 1-m \right)\times{joint effect \mathrm{of}S_{0}}_{Anc2} (6)$$

where $m$ indicates the admixed proportion of ancestry 1 at the genome level, and $(1-m)$indicates the admixed proportion of ancestry 2. Following the previous analysis, we selected ancestral source populations, EAS and EUR, as references to test the admixture effects in XJU. To avoid the batch effects of gene expression, eQTLs were considered as $S_{0}$, and the ES of the eQTLs was considered as the joint effects of variants and ancestral backgrounds as the potential causal factors. We analyzed the covariance between XJU and its ancestral source populations to test whether the change in ES was in the ideal state. When *P* < 0.05, the ES changes were not in the ideal state, and we further compared the degree of change (beta value) via the linear regression model in the R package “MatrixEQTL” to determine the direction of the change in ES. Forty individuals were randomly sampled from each group to guarantee a comparable sample size.

Supplementary Figures

Fig. S1 | The distribution of successful mapping rates of XJU.

The mapping ratio of each sample was listed in Table S1.


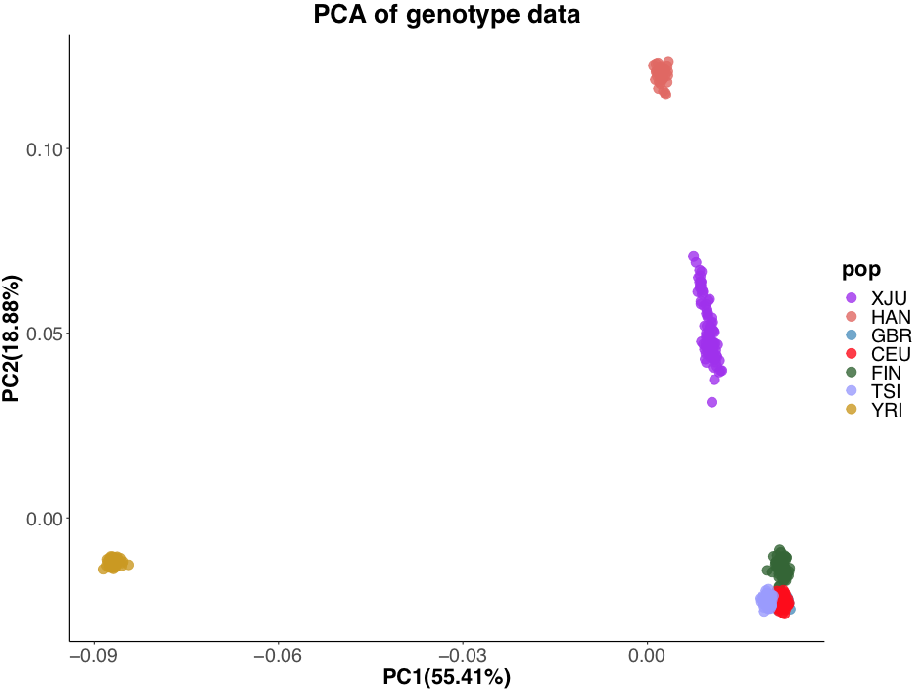


Fig. S2 | Principal component analysis (PCA) for XJU, east and west Eurasian in DNA level.

The x-axis and y-axis indicated the first and second principal components of PCA. PCA was applied with smartpca [1, 2] using 4,772,642 autosome biallelic SNPs (MAF > 0.01). The overlapped dots of GBR and CEU indicated the similarity of these two groups in genetics.


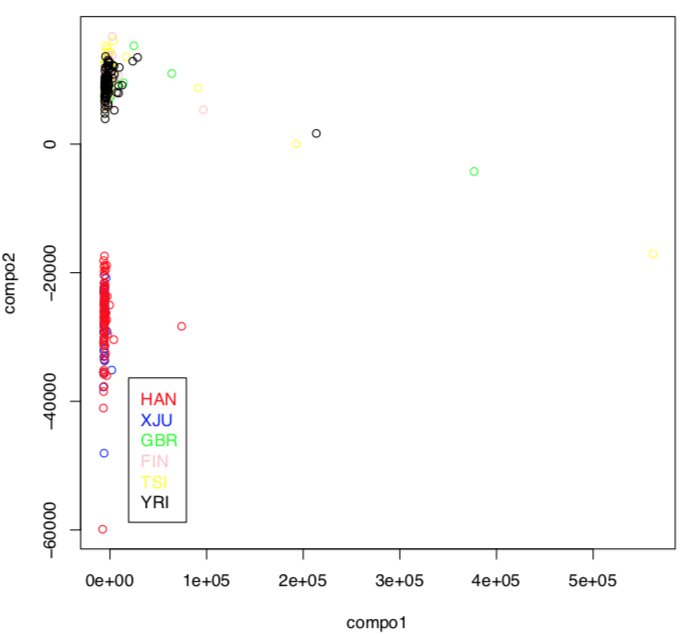


Fig. S3 | The PCA plot of gene expression.

Samples were from the Genetic European Variation in Health and Disease (GEUVADIS) project [21, 22, 39] and our dataset. Each circle indicated one sample. The populations of HAN and XJU were collected by ours, and the main population samples were collected the by GEUVADIS project. There were obvious batch effects between the two different datasets.

Fig. S4 | PCA for XJU, east and west Eurasian, and other populations in gene expression level.

Only autosome genes were considered. The figures shown in the first row were PCA for all expressed genes. In total, there were 21,008 autosome genes. When two datasets (our dataset and the GEUVADIS dataset) were combined, the batch effect could be observed. In the GEUVADIS dataset, a slight batch effect could also be observed. The figures shown in the second row were PCA for all expressed genes with PEER [17]. The hidden factor was set as 3. The batch effect could be observed between datasets but not within each dataset. HAN and GBR were referred to EAS and EUR respectively as ancestral source populations of XJU.

Fig S5 | PCA for XJU, east and west Eurasian, and other populations in AS events level.

The red circles indicated the subgroup in datasets, reflecting the batch effect. The x-axis and y-axis indicated the first and second principal components. Autosome AS events were considered. PCA was conducted in each type of AS (SE, IR, A5SS, A3SS), including 47,873 SE events, 2,079 IR events, 2,106 A5SS events, and 3,027 A3SS events. In this panel, each column was shown as one type of AS, flowed by SE, IR, A5SS, and A3SS. (A-D) PCA of XJU, east and west Eurasian. Batch effects were observed between the two datasets. And within the GEUVADIS dataset, samples generated by lab 2 were clustered (in red circle). (E-H) PCA of XJU and EAS. There were no batch effects observed, of which samples were generated in the same batch. (I-L) PCA of West Eurasian. In the GEUVADIS dataset, a batch effect existed: samples generated by lab 2 were clustered (in red circle) as a subgroup. HAN and GBR were referred to EAS and EUR respectively as ancestral source populations of XJU.

Fig S6 | PCA for XJU, east and west Eurasian, and other populations in AS data after the subgroup was removed.

West Eurasian in GEUVADIS dataset generated from lab 2 were removed. Autosome AS events was only considered. PCA was conducted in 4 types of AS (SE, IR, A5SS, A3SS) respectively, including 47,873 SE events, 2,079 IR events, 2,106 A5SS events, and 3,027 A3SS events. (A-D) PCA of XJU, east and west Eurasian. Batch effects were still observed as two different datasets. (E-H) PCA of West Eurasian. In the GEUVADIS dataset, the batch effect was removed when samples generated by lab 2 were removed. These samples were also removed in our downstream analysis. HAN and GBR were referred to EAS and EUR respectively as ancestral source populations of XJU.

Fig S7 | PCA for XJU, east and west Eurasian, and other populations in transcriptome level with AS data after the subgroup was removed and PEER normalization.

West Eurasian in GEUVADIS dataset generated from lab 2 were removed. Autosome AS events was only considered. PCA was conducted in 4 types of AS respectively. PEER was applied to relieve the batch effect caused by hidden factors (including populations, sex, generated labs, and age) in two datasets jointly and respectively. Each column indicated one type of AS: SE, IR, A5SS, and A3SS. (A-D) PCA of XJU, east and west Eurasian. The batch effect was only observed in the type of SE between the two datasets. (E-H) PCA of XJU and EAS. We did not find any batch effects. (I-L) PCA of West Eurasian. We did not find any batch effects. The information on sample size applied in the downstream analysis was shown in Table S2. HAN and GBR were referred to EAS and EUR respectively as ancestral source populations of XJU.


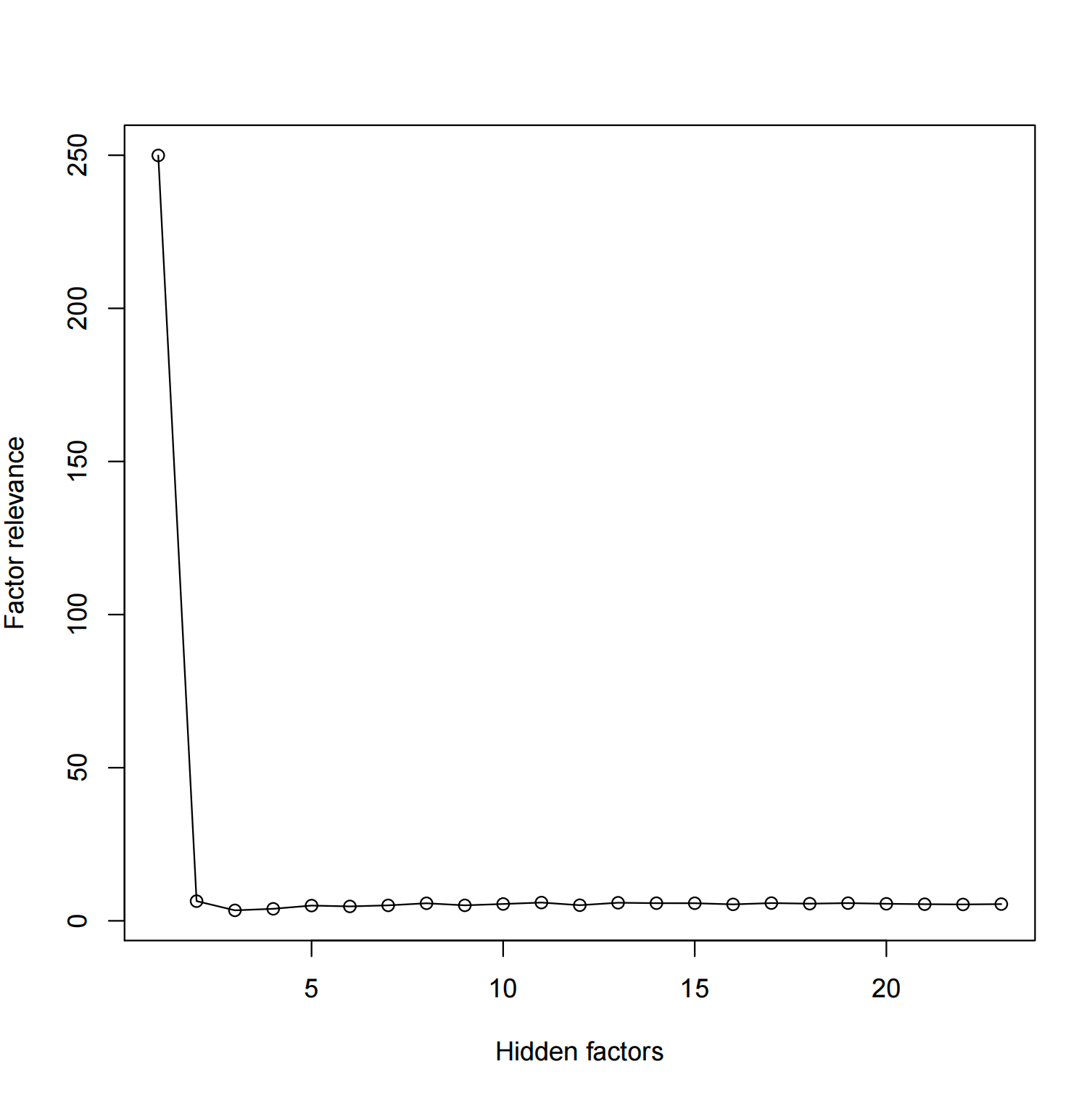


Fig S8 | Diagnostic plot of the hidden factor relevance (ARD parameters) in PEER

To determine the appropriate number of hidden factors required in the PEER analysis in the expression profiles, we first set a sufficiently large value of 23 and found that only the first hidden factor was with significantly high accuracy (measured by variance), which was consistent with the reliable quality of our sequencing data and with the results in the PEER protocol [40]. We kept the hidden factors at the recommended value of 3 to avoid overfitting and to save computing time. This analysis was conducted with XJU transcriptome data.

Fig S9 | The functional elements enrichment of HDSs in XJU.

The functional enrichment analysis was conducted with Ensembl Regulatory Build and coding annotations. The y-axis denoted the odds ratio of the enrichment between HDSs and the null in each category, denoting the degree of enrichment. The red line was considered as a reference to null distribution (OR = 1). The number of * suggested the significance measured by adjusted *P* value in Fisher’s exact test. * for *P* < 0.05, ** for *P* < 0.01, *** for *P* < 0.001.

Fig S10 | The GWAS enrichment of HDSs in XJU.

The GWAS enrichment analysis was conducted with GWAS Category (Methods). The enrichments were conducted under each threshold per trait. The x-axis indicated the conducted threshold in corresponding GWASs. The size of the circles indicated the odds ratio of enrichments. The color of the circles indicates the significance level measured by the adjusted *P* value.

Fig S11 | The GWAS enrichment of HDSs-covered genes in XJU.

Fig S12 | The KEGG enrichment of HDSs-covered genes in XJU.


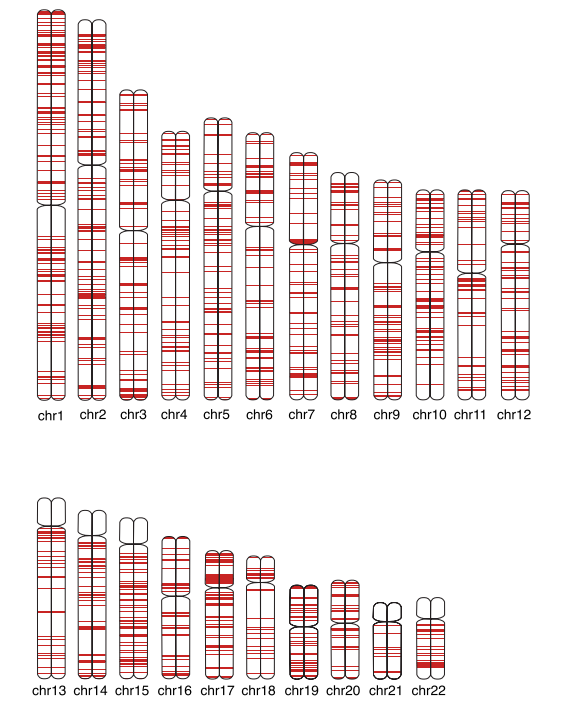


Fig S13 | The distribution of HDGs in XJU.

The HDGs were exhibited in each chromosome by Rishishwar, Conley [41].

Fig S14 | The GWAS enrichment of HDGs in XJU.


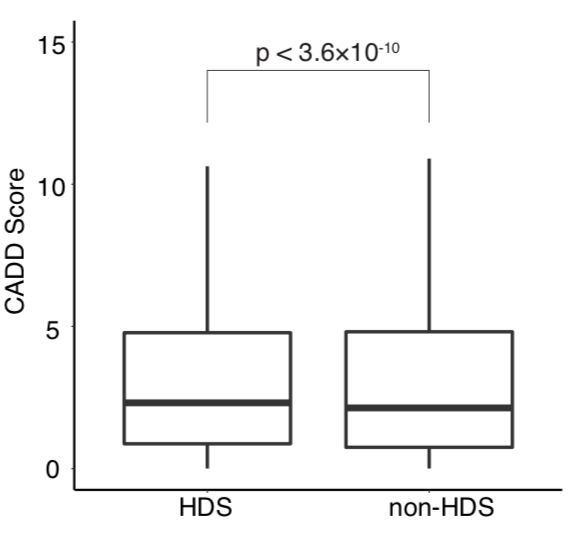


Fig S15 | The CADD comparison between HDSs and non-HDSs in XJU. A Student *t* test was conducted to compare the differences.


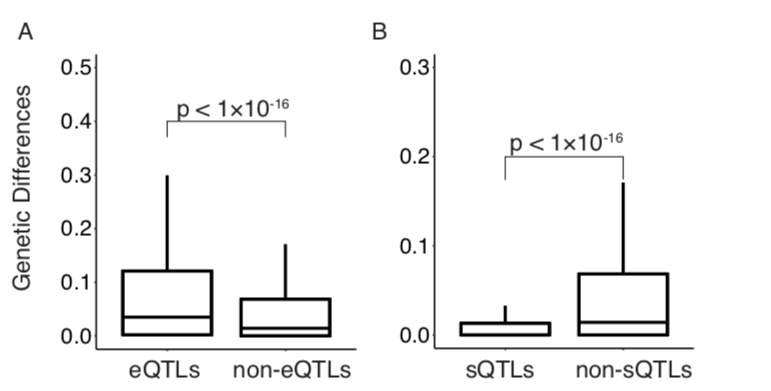


Fig S16 | The comparison of ancestral genetic differences between eQTLs and non-eQTLs

(A) sQTLs and non-sQTLs (B) respectively. We used a one-tailed *t* test to conduct comparisons. The LD structure was considered in permutation tests. We conducted random sampling 100 times to assure the results with the same significance level (*P* < 10^−16^)


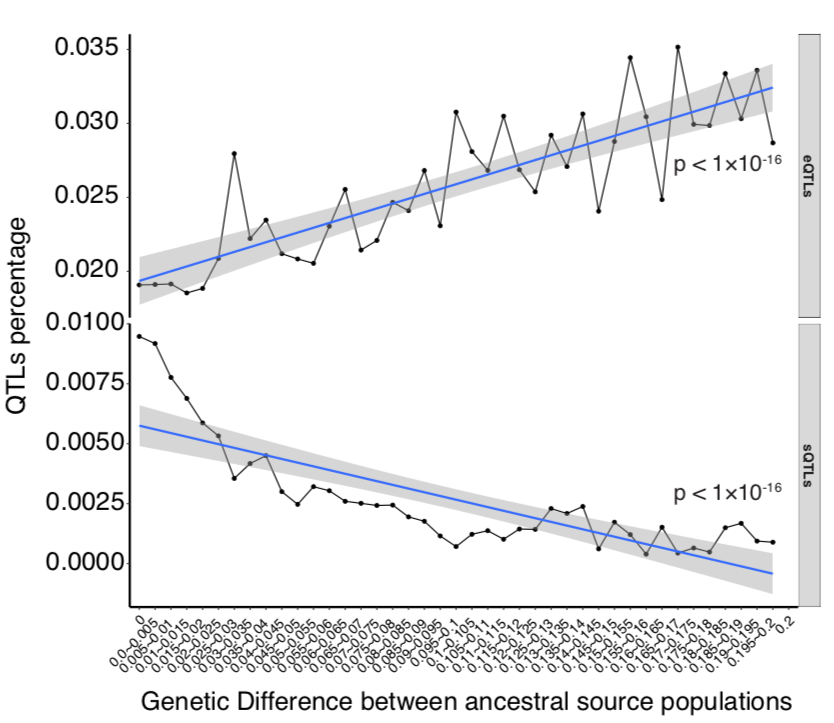


Fig S17 | The distribution of ancestral genetic difference vs. QTLs percentages in XJU.

Whole genome SNPs were divided into 40 blocks by increased ancestral genetic difference (step = 0.005,$F_{ST}$between EAS and EUR). A SNP is labeled as “0” when the allele difference between ancestral populations is equal to or less than 0; a SNP is labeled as “0.2” when the allele difference between ancestral populations is equal to or larger than 0.2. We measured the tendency of QTLs proportions with increased ancestral genetic difference. The ancestral genetic difference of XJU SNPs was calculated with the allele frequency of sites in ancestral source populations of XJU. There was no difference among the LD value of QTLs detected across different F_ST_ bins both in eQTLs (ANOVA, *P* = 0.653) and sQTLs (ANOVA, *P* = 0.433).


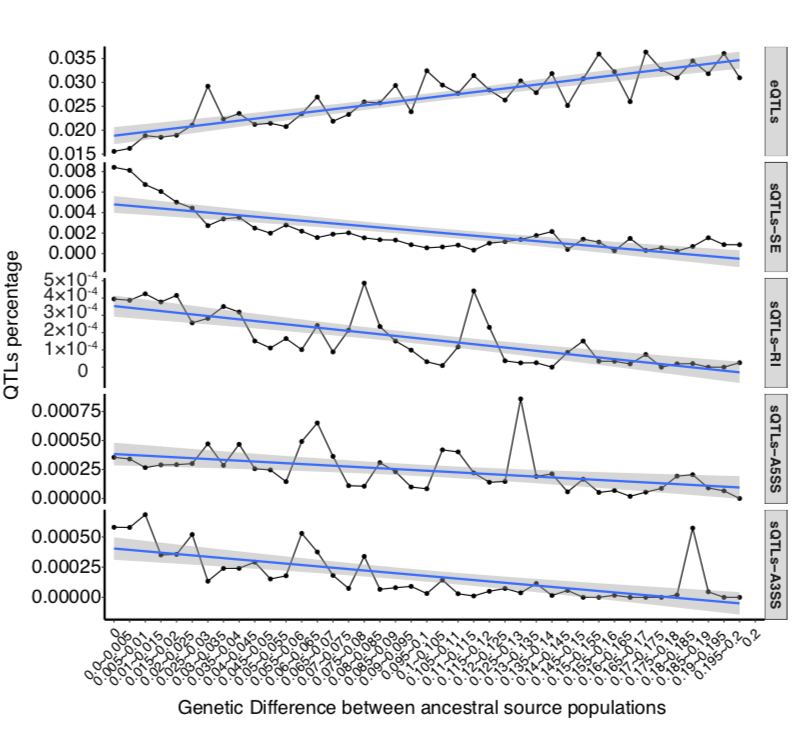


Fig S18 | The distribution of ancestral genetic difference vs. QTLs percentages in XJU.

The sQTLs detected in different AS types were measured respectively.

Fig S19 | The functional elements enrichment of HDS-eQTLs in XJU.

The background SNPs applied in the enrichment analysis were narrowed down to the *cis* eQTLs’ regions. The number of * suggested the significance measured by adjusted *P* value in Fisher’s exact test. * *P* < 0.05, ** *P* < 0.01, *** for *P* < 0.001.

Fig S20 | The functional elements enrichment of HDS-sQTLs in XJU.

The background SNPs applied in the enrichment analysis were narrowed down to the *cis* sQTLs’ regions. The number of * suggested the significance measured by adjusted *P* value in Fisher’s exact test. * *P* < 0.05, ** *P* < 0.01, *** *P* < 0.001. NS, not significant.

Fig S21 | The GWAS enrichment of HDS-eQTLs in XJU.

Fig S22 | The GWAS enrichment of HDS-sQTLs in XJU.

Fig S23 | The functional elements enrichment of HDS-ASE in XJU.

The background SNPs applied in the enrichment analysis were narrowed down to the coding regions. The number of * suggested the significance measured by adjusted *P* value in Fisher’s exact test. * *P* < 0.05, ** *P* < 0.01, *** *P* < 0.001.

Fig S24 | The GWAS enrichment of HDS-ASE in XJU.

Fig S25 | The KEGG enrichment of HDS-aseGenes in XJU.

Fig S26 | The GWAS enrichment of HDS-aseGenes in XJU.


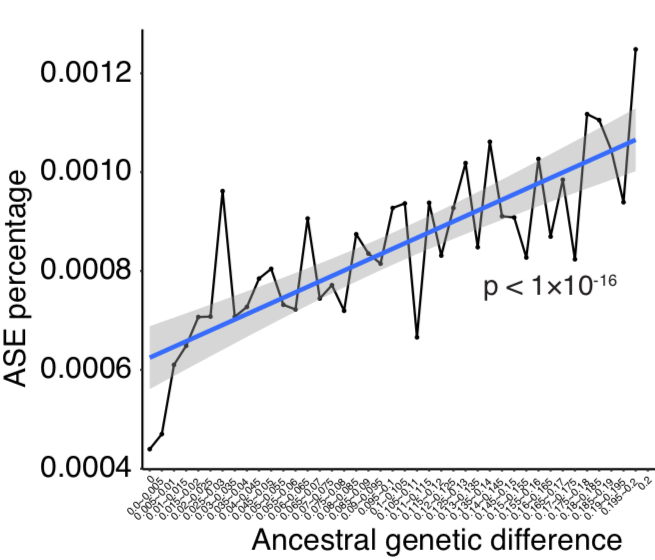


Fig S27 | The distribution of ancestral genetic difference vs. ASE percentages in XJU.


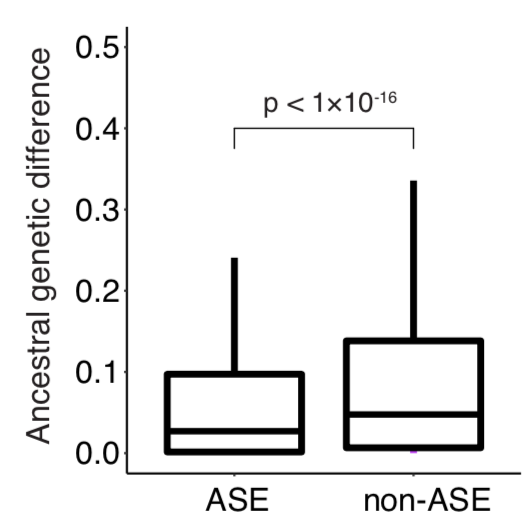


Fig S28 | The comparison of ancestral genetic differences between ASE and non-ASE with *t* test.


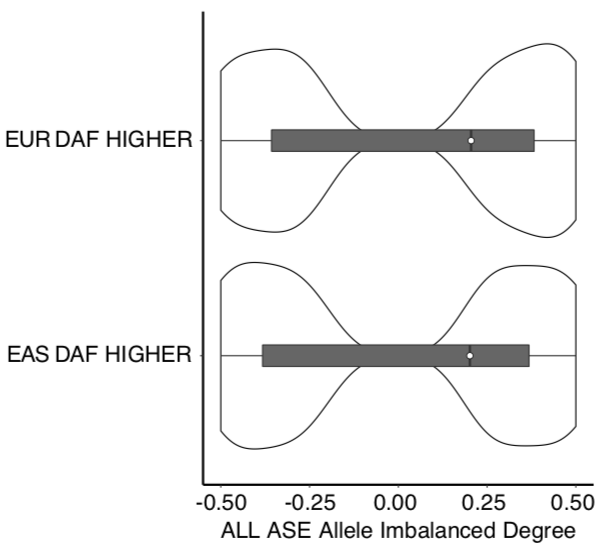


Fig S29 | The comparison of the degree of allelic imbalanced expression (AI) among all ASE.

All ASE were divided into two groups by derived allele frequency (DAF). If the allele frequency of the derived allele of ASE was higher in EUR, the allele would be regarded as the Western-origin allele with a high possibility. While if the allele frequency of the derived allele of ASE was higher in EAS, the allele would be regarded as the Eastern-origin allele with a high possibility. The x-axis indicated the AI levels. The “+” indicated the derived alleles were highly expressed and the “-” indicated the ancestry alleles were highly expressed.

Fig S30 | The functional elements enrichment of HDS-aseQTLs in XJU.

The background SNPs applied in the enrichment analysis were narrowed down to the *cis* QTLs regions. The number of * suggested the significance measured by adjusted *P* value in Fisher’s exact test. * *P* < 0.05, ** *P* < 0.01, *** *P* < 0.001. NS, not significant.


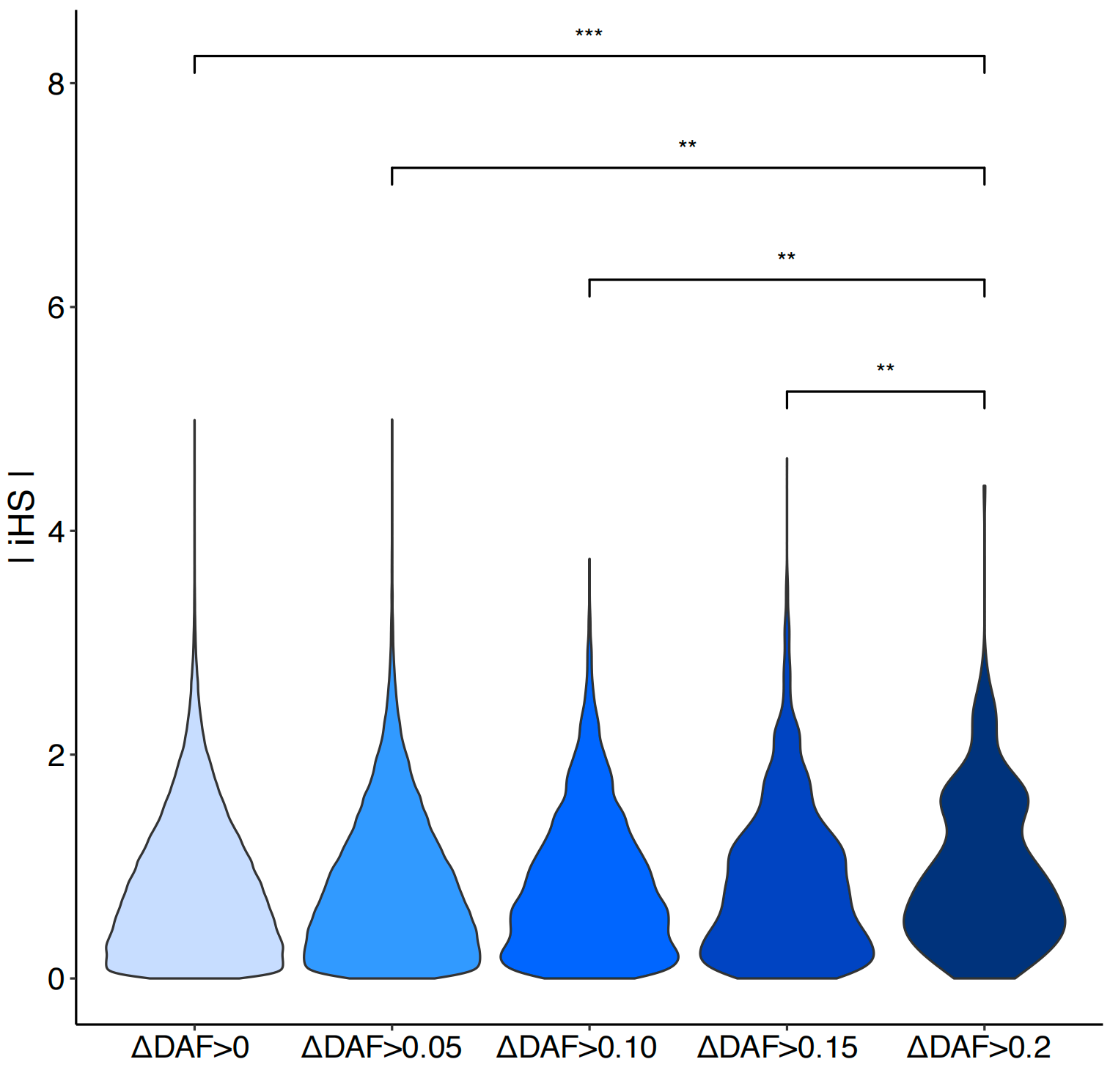


Fig S31 | HDSs with a higher degree of deviation indicated evidence of adaptation.

One-tailed *t* tests were applied on |iHS| of the variants across different deviation categories. The number of * suggested the significance measured by adjusted *P* value. * *P* < 0.05, ** *P* < 0.01, *** *P* < 0.001.


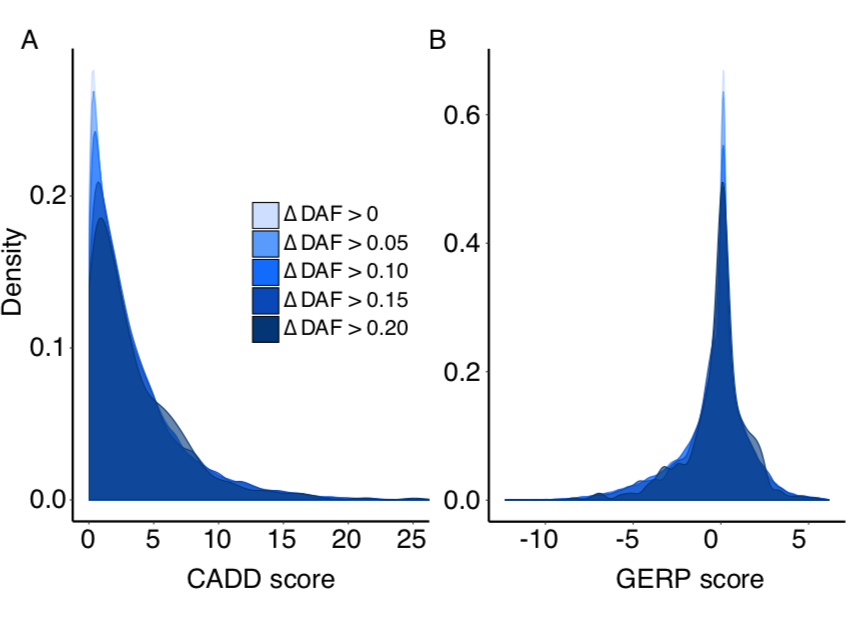


Fig S32 | The density plots of CADD and GERP scores of HDSs conditioned on the degree of the deviation.

The HDSs were divided by the deviation of the derived allele frequency between the observed and expected derived allele frequency in XJU (Methods). The color indicated the deviation degree, and the x-axis indicated the CADD scores (A) and GERP scores (B).


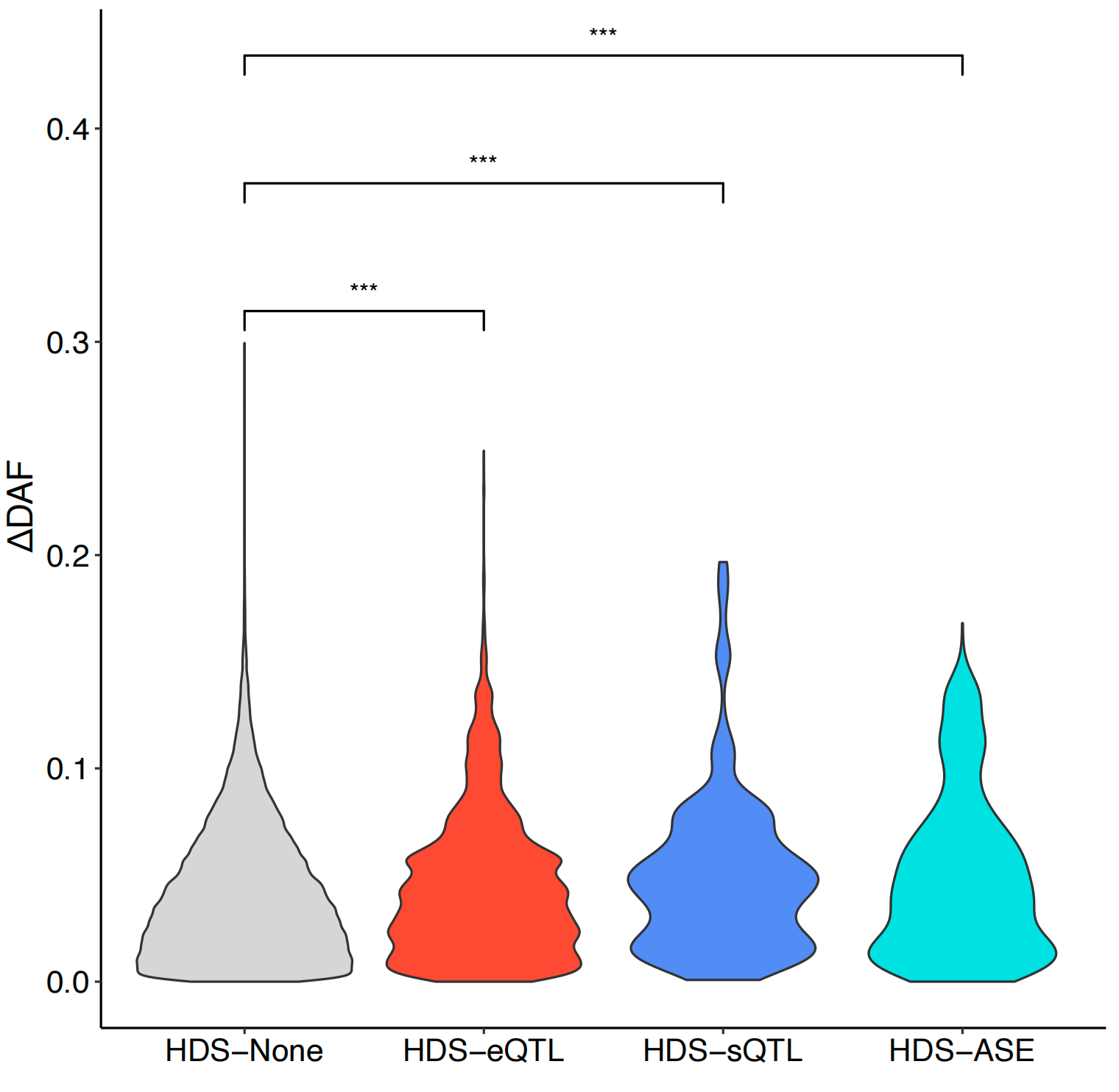


Fig S33 | HDSs with putative regulatory functions (HDS-QTLs and HDS-ASE) showed a higher degree of deviation compared with HDSs without putative regulatory functions.

One-tailed *t* tests were applied on |ΔDAF| of potentially functional variants (eQTLs, sQTLs, and ASE) and the variants without putative functions. The number of * suggested the significance measured by adjusted *P* value. * *P* <0.05, ** *P* <0.01, *** *P* <0.001.

Fig S34 | The functional elements enrichment of the HDSs with multiple putative regulatory functions and possibly having undergone harder selective pressure in XJU.

The background SNPs were narrowed down to the HDSs with no putative regulatory functions (not QTLs or ASE). The number of * suggested the significance measured by adjusted *P* value. * *P* < 0.05, ** *P* < 0.01, *** *P* < 0.001. NS, not significant.

Fig S35 | The distribution of effect size of all eQTLs in XJU.

All of the eQTLs were categorized by ancestral genetic differences as the x-axis labeled. A SNP is labeled as “0” when the allele difference between ancestral populations is equal to or less than 0; a SNP is labeled as “0.2” when the allele difference between ancestral populations is equal to or larger than 0.2.

Fig S36 | The KEGG pathway network of HDSE-gene in XJU.

We used this pathway network to illustrate the relationship between enriched genes and pathways. If there is an overlap between the different genes of the two pathways, it means that there is an overlapping relationship between the two nodes, which are connected by lines. Each dot indicated one enriched pathway of HDSE-eGenes. The color of each dot indicated the enrichment degree, and the size of each dot indicated the number of enriched HDSE-eGenes.

Fig S37 | The schematic diagram of aseQTLs of two ASEs on *UTS2.*

The aseQTL rs664689 is associated with the two ASEs, rs2890565 and rs228648 in XJU. When the aseQTL was heterozygous, ASE could be observed as imbalanced expressed.


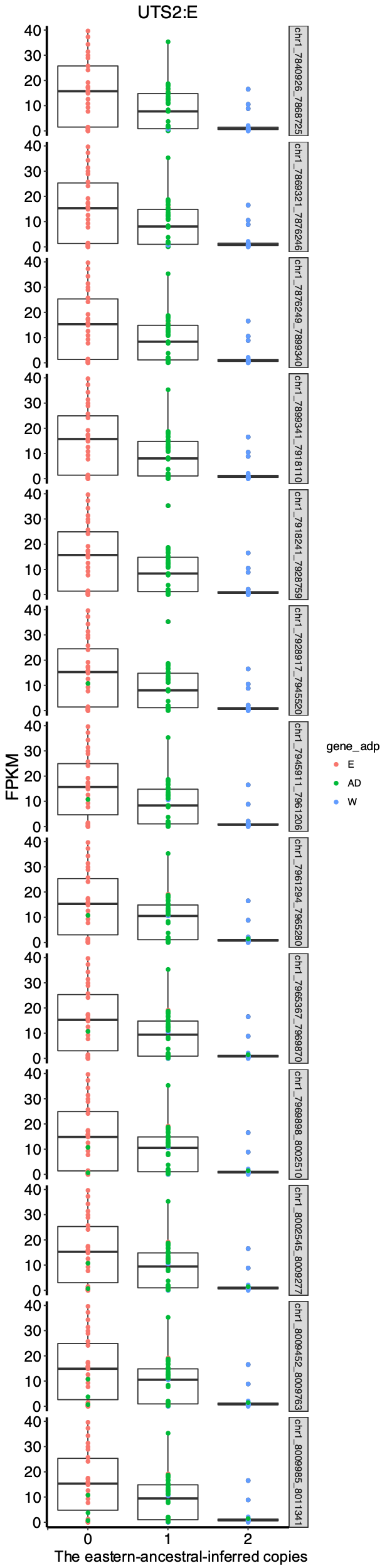


Fig S38 | The associated ancestral inferred segments of XJU.

Each dot indicated one sample. The x-axis indicated the samples grouped by the copy numbers of the ancestral-inferred segments. The “0” indicated 0 copies of the Eastern-ancestral-inferred segments, the “1” indicated 1 copy of the Eastern-ancestral-inferred segments, and the “2” indicated 2 copies of the Eastern-ancestral-inferred segments. The y-axis indicated the expression levels of *UTS2*. The color of the dots indicated the ancestral inference of the gene *UTS2*. The red color indicated the gene was inferred as derived from Eastern ancestry, the blue indicated the gene was inferred as derived from Western ancestry, and the green indicated the gene was inferred as admixed with Eastern and Western ancestries (Methods). There were 13 inferred segments related to *UTS2*.


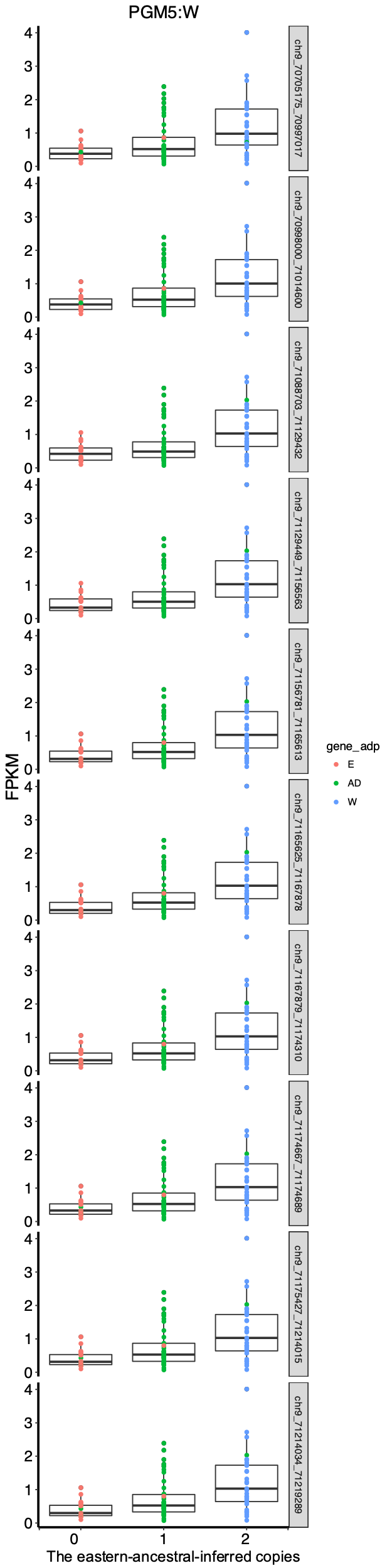


Fig S39 | The associated ancestral inferred segments of XJU.

Each dot indicated one sample. The x-axis indicated the samples grouped by the copy numbers of the ancestral-inferred segments. The y-axis indicated the expression levels of *PGM5*. The color of the dots indicated the ancestral inference of the gene *PGM5*. There were 10 inferred segments related to *PGM5*.

Fig S40 | The KEGG enrichment of the associated genes modeled in the ideal state.

Fig S41 | The KEGG enrichment of the associated genes modeled in the specific state (relaxed or enhanced).


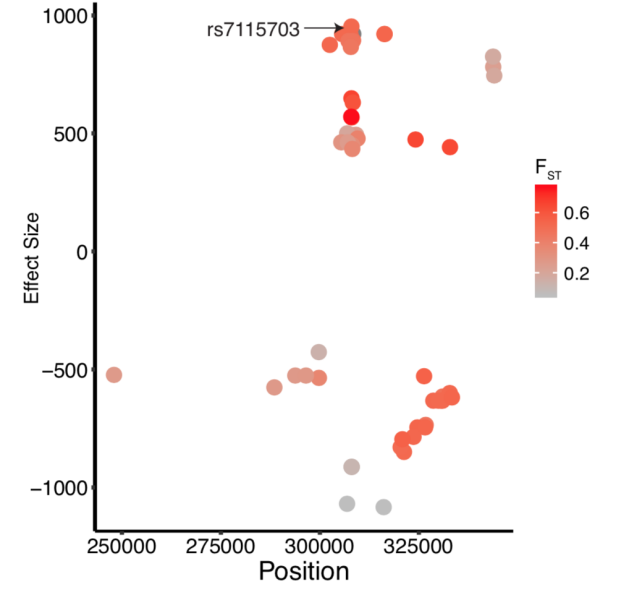


Fig S42 | The 56 eQTLs of *IFITM3* in XJU.


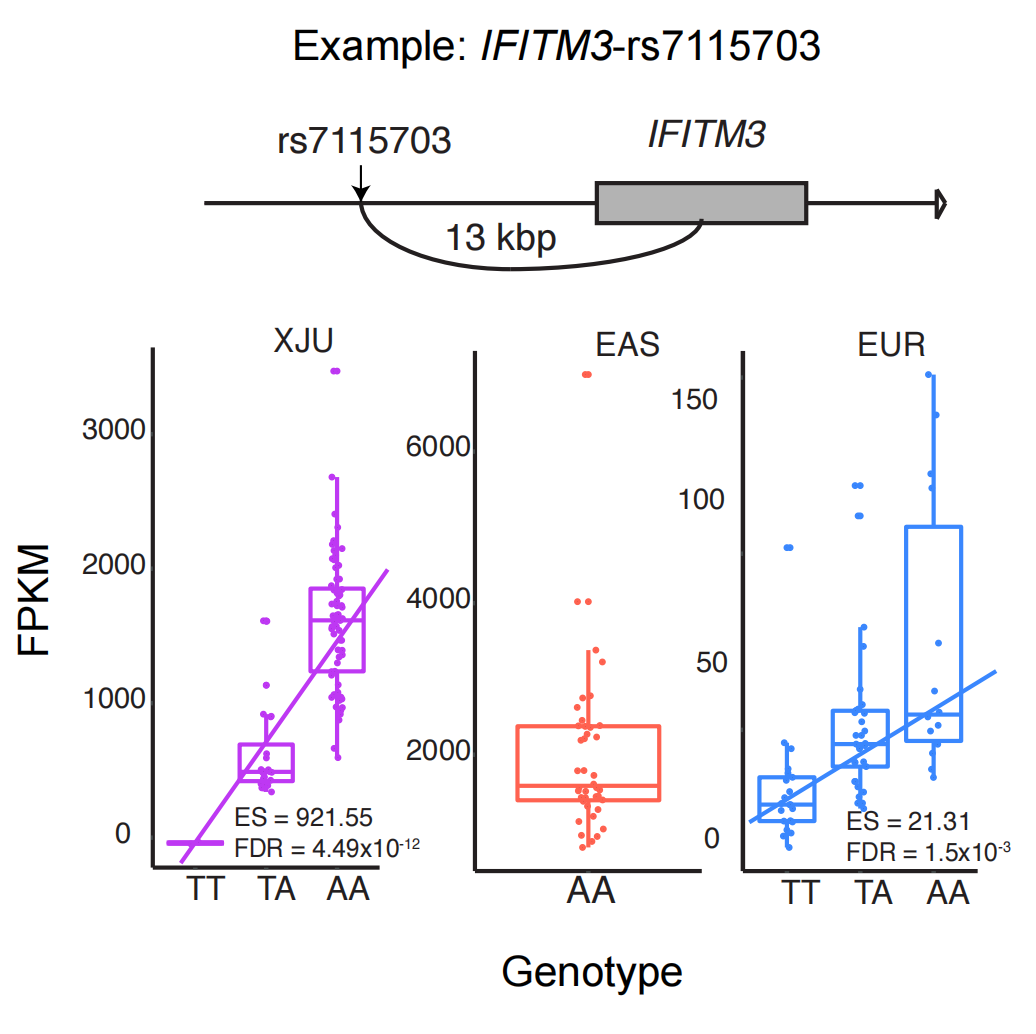


Fig S43 | The associations between rs711570 and *IFITM3* in XJU, EAS, and EUR.

The x-axis indicated the genotypes.

Supplementary Tables

Table S1 | The sequencing properties of XJU and HAN per individual.

The table showed the summary data about sequences for each individual, including the information on the total sequenced reads, mapped reads, the average mapped length of reads for exon regions, mapping ratio, and estimated coverage ratio for the exon regions.

| Sample ID | Total Reads | Mapped Reads | Average Mapped Length  (exon regions) | Mapping Ratio | Estimated Coverage  (exon regions) |
| --- | --- | --- | --- | --- | --- |
| XJU S1 | 10,251,795 | 9,475,207 | 193.67 | 92.42% | 26.42 |
| XJU S2 | 11,738,323 | 10,889,341 | 193.76 | 92.77% | 30.38 |
| XJU S3 | 12,270,797 | 11,348,181 | 193.64 | 92.48% | 31.64 |
| XJU S4 | 12,171,177 | 11,257,833 | 193.65 | 92.50% | 31.39 |
| XJU S5 | 11,623,878 | 10,757,291 | 193.64 | 92.54% | 29.99 |
| XJU S6 | 11,497,744 | 10,621,865 | 193.62 | 92.38% | 29.61 |
| XJU S7 | 11,404,563 | 10,553,477 | 193.66 | 92.54% | 29.42 |
| XJU S8 | 12,227,677 | 11,307,332 | 193.55 | 92.47% | 31.51 |
| XJU S9 | 12,127,497 | 11,215,619 | 193.61 | 92.48% | 31.26 |
| XJU S10 | 12,249,004 | 11,346,649 | 193.65 | 92.63% | 31.63 |
| XJU S11 | 11,044,696 | 10,175,454 | 193.59 | 92.13% | 28.36 |
| XJU S12 | 11,681,898 | 10,809,499 | 193.6 | 92.53% | 30.13 |
| XJU S13 | 12,157,475 | 11,213,290 | 193.56 | 92.23% | 31.25 |
| XJU S14 | 10,589,438 | 9,755,824 | 193.64 | 92.13% | 27.2 |
| XJU S15 | 10,599,476 | 9,715,671 | 193.38 | 91.66% | 27.05 |
| XJU S16 | 11,900,734 | 11,011,575 | 193.43 | 92.53% | 30.67 |
| XJU S17 | 11,431,079 | 10,541,321 | 193.22 | 92.22% | 29.32 |
| XJU S18 | 11,735,423 | 10,797,885 | 193.37 | 92.01% | 30.06 |
| XJU S19 | 12,217,834 | 11,318,962 | 193.38 | 92.64% | 31.51 |
| XJU S20 | 11,725,380 | 10,846,880 | 193.31 | 92.51% | 30.19 |
| XJU S21 | 11,516,740 | 10,656,643 | 193.39 | 92.53% | 29.67 |
| XJU S22 | 11,975,877 | 11,066,513 | 193.37 | 92.41% | 30.81 |
| XJU S23 | 11,073,929 | 10,067,831 | 193.4 | 90.91% | 28.03 |
| XJU S24 | 12,306,604 | 11,322,833 | 193.55 | 92.01% | 31.55 |
| XJU S25 | 11,970,029 | 11,015,845 | 193.57 | 92.03% | 30.7 |
| XJU S26 | 13,477,510 | 12,367,603 | 193.55 | 91.76% | 34.46 |
| XJU S27 | 13,074,153 | 12,117,312 | 193.54 | 92.68% | 33.76 |
| XJU S28 | 11,906,039 | 11,043,174 | 193.54 | 92.75% | 30.77 |
| XJU S29 | 12,547,334 | 11,622,089 | 193.56 | 92.63% | 32.39 |
| XJU S30 | 11,587,863 | 10,708,645 | 193.61 | 92.41% | 29.85 |
| XJU S31 | 11,066,501 | 10,256,242 | 193.58 | 92.68% | 28.58 |
| XJU S32 | 11,315,975 | 10,495,243 | 193.55 | 92.75% | 29.25 |
| XJU S33 | 10,572,378 | 9,731,723 | 193.61 | 92.05% | 27.13 |
| XJU S34 | 11,245,654 | 10,442,082 | 193.53 | 92.85% | 29.09 |
| XJU S35 | 11,836,195 | 10,982,968 | 193.64 | 92.79% | 30.62 |
| XJU S36 | 11,326,762 | 10,492,792 | 193.7 | 92.64% | 29.26 |
| XJU S37 | 13,163,271 | 12,212,521 | 194.16 | 92.78% | 34.14 |
| XJU S38 | 13,154,939 | 12,224,118 | 194.23 | 92.92% | 34.18 |
| XJU S39 | 13,750,456 | 12,813,643 | 194.33 | 93.19% | 35.85 |
| XJU S40 | 13,387,932 | 12,492,785 | 194.34 | 93.31% | 34.95 |
| XJU S41 | 13,117,225 | 12,218,523 | 194.36 | 93.15% | 34.19 |
| XJU S42 | 13,229,063 | 12,303,326 | 194.37 | 93.00% | 34.43 |
| XJU S43 | 12,494,594 | 11,565,895 | 194.41 | 92.57% | 32.37 |
| XJU S44 | 13,241,353 | 12,257,446 | 194.4 | 92.57% | 34.31 |
| XJU S45 | 12,758,380 | 11,757,487 | 194.44 | 92.16% | 32.91 |
| XJU S46 | 14,196,928 | 13,047,018 | 194.42 | 91.90% | 36.52 |
| XJU S47 | 10,927,694 | 9,960,053 | 194.34 | 91.15% | 27.87 |
| XJU S48 | 12,790,255 | 11,892,117 | 194.41 | 92.98% | 33.29 |
| XJU S49 | 13,082,324 | 12,153,243 | 194.38 | 92.90% | 34.01 |
| XJU S50 | 12,363,475 | 11,450,465 | 194.35 | 92.62% | 32.04 |
| XJU S51 | 12,514,845 | 11,617,714 | 194.25 | 92.83% | 32.49 |
| XJU S52 | 12,346,876 | 11,433,809 | 194.24 | 92.60% | 31.97 |
| XJU S53 | 12,144,310 | 11,161,033 | 194.3 | 91.90% | 31.22 |
| XJU S54 | 13,284,270 | 12,387,934 | 194.24 | 93.25% | 34.64 |
| XJU S55 | 13,061,434 | 12,180,802 | 194.34 | 93.26% | 34.08 |
| XJU S56 | 10,231,741 | 9,474,463 | 194.34 | 92.60% | 26.51 |
| XJU S57 | 12,908,394 | 11,986,447 | 194.24 | 92.86% | 33.52 |
| XJU S58 | 12,600,910 | 11,603,790 | 194.23 | 92.09% | 32.45 |
| XJU S59 | 12,894,876 | 12,052,485 | 194.21 | 93.47% | 33.7 |
| XJU S60 | 12,551,842 | 11,656,049 | 194.24 | 92.86% | 32.6 |
| XJU S61 | 12,244,998 | 11,361,620 | 194.14 | 92.79% | 31.76 |
| XJU S62 | 11,903,567 | 11,022,929 | 194.19 | 92.60% | 30.82 |
| XJU S63 | 14,198,055 | 13,186,100 | 194.06 | 92.87% | 36.84 |
| XJU S64 | 14,051,558 | 13,071,439 | 194.06 | 93.02% | 36.52 |
| XJU S65 | 14,517,998 | 13,472,991 | 194.09 | 92.80% | 37.65 |
| XJU S66 | 12,887,810 | 11,975,821 | 194.04 | 92.92% | 33.46 |
| XJU S67 | 14,315,571 | 13,282,497 | 194.04 | 92.78% | 37.11 |
| XJU S68 | 14,297,492 | 13,282,267 | 194.03 | 92.90% | 37.1 |
| XJU S69 | 13,137,859 | 12,220,669 | 194.07 | 93.02% | 34.15 |
| XJU S70 | 14,380,867 | 13,340,404 | 194.05 | 92.76% | 37.27 |
| XJU S71 | 12,927,187 | 12,023,544 | 193.97 | 93.01% | 33.58 |
| XJU S72 | 12,819,852 | 11,911,324 | 194.03 | 92.91% | 33.27 |
| XJU S73 | 12,815,527 | 11,896,065 | 194.1 | 92.83% | 33.24 |
| XJU S74 | 14,002,203 | 12,988,263 | 194.1 | 92.76% | 36.3 |
| XJU S75 | 11,438,261 | 10,638,226 | 194.02 | 93.01% | 29.72 |
| XJU S76 | 13,901,543 | 12,847,735 | 194.13 | 92.42% | 35.91 |
| XJU S77 | 14,305,778 | 13,261,757 | 194.07 | 92.70% | 37.05 |
| XJU S78 | 15,031,265 | 13,975,956 | 193.98 | 92.98% | 39.03 |
| XJU S79 | 13,205,973 | 12,245,383 | 194.04 | 92.73% | 34.21 |
| XJU S80 | 12,933,566 | 11,967,752 | 193.94 | 92.53% | 33.42 |
| XJU S81 | 13,989,565 | 12,981,812 | 193.91 | 92.80% | 36.24 |
| XJU S82 | 14,171,447 | 13,117,987 | 194.1 | 92.57% | 36.66 |
| XJU S83 | 13,941,157 | 12,893,907 | 194.05 | 92.49% | 36.02 |
| XJU S84 | 13,272,469 | 12,165,288 | 194.09 | 91.66% | 33.99 |
| XJU S85 | 12,948,481 | 11,548,841 | 194.06 | 89.19% | 32.27 |
| XJU S86 | 14,317,818 | 13,169,359 | 194.02 | 91.98% | 36.79 |
| XJU S87 | 12,831,739 | 11,832,458 | 193.99 | 92.21% | 33.05 |
| XJU S88 | 13,138,728 | 12,217,230 | 194.07 | 92.99% | 34.14 |
| XJU S89 | 13,256,957 | 12,298,605 | 194.01 | 92.77% | 34.35 |
| XJU S90 | 12,278,291 | 11,319,735 | 194.13 | 92.19% | 31.64 |
| HAN S1 | 11,028,938 | 10,181,822 | 193.58 | 92.32% | 28.38 |
| HAN S2 | 12,166,820 | 11,189,133 | 193.55 | 91.96% | 31.18 |
| HAN S3 | 10,897,603 | 9,130,181 | 193.25 | 83.78% | 25.4 |
| HAN S4 | 12,589,279 | 11,593,443 | 193.57 | 92.09% | 32.31 |
| HAN S5 | 12,230,928 | 11,342,813 | 193.56 | 92.74% | 31.61 |
| HAN S6 | 10,810,716 | 9,940,022 | 193.54 | 91.95% | 27.7 |
| HAN S7 | 12,345,861 | 11,498,232 | 193.45 | 93.13% | 32.02 |
| HAN S8 | 11,801,723 | 10,862,226 | 193.5 | 92.04% | 30.26 |
| HAN S9 | 12,127,427 | 11,258,754 | 193.52 | 92.84% | 31.37 |
| HAN S10 | 13,336,125 | 12,324,801 | 193.55 | 92.42% | 34.34 |
| HAN S11 | 11,922,355 | 11,084,957 | 193.47 | 92.98% | 30.88 |
| HAN S12 | 12,079,047 | 11,166,470 | 193.59 | 92.44% | 31.12 |
| HAN S13 | 12,281,431 | 11,276,881 | 193.53 | 91.82% | 31.42 |
| HAN S14 | 12,287,299 | 11,518,263 | 193.5 | 93.74% | 32.09 |
| HAN S15 | 11,516,314 | 10,655,854 | 193.45 | 92.53% | 29.68 |
| HAN S16 | 11,457,007 | 10,660,189 | 193.52 | 93.05% | 29.7 |
| HAN S17 | 10,834,876 | 9,994,620 | 193.36 | 92.24% | 27.82 |
| HAN S18 | 11,745,598 | 10,822,146 | 193.46 | 92.14% | 30.14 |
| HAN S19 | 11,011,734 | 10,205,498 | 193.52 | 92.68% | 28.43 |
| HAN S20 | 10,079,482 | 9,267,141 | 193.4 | 91.94% | 25.8 |
| HAN S21 | 11,422,324 | 10,456,625 | 193.55 | 91.55% | 29.14 |
| HAN S22 | 12,160,392 | 11,239,082 | 193.52 | 92.42% | 31.31 |
| HAN S23 | 12,122,491 | 11,195,552 | 193.49 | 92.35% | 31.19 |
| HAN S24 | 11,524,616 | 10,635,293 | 193.62 | 92.28% | 29.65 |
| HAN S25 | 11,287,144 | 10,413,501 | 193.4 | 92.26% | 29 |
| HAN S26 | 10,621,541 | 9,569,096 | 193.78 | 90.09% | 26.7 |
| HAN S27 | 12,244,136 | 11,311,472 | 193.62 | 92.38% | 31.53 |
| HAN S28 | 11,306,225 | 10,447,127 | 193.57 | 92.40% | 29.11 |
| HAN S29 | 12,249,430 | 11,307,773 | 193.57 | 92.31% | 31.51 |
| HAN S30 | 11,324,792 | 10,485,615 | 193.5 | 92.59% | 29.21 |
| HAN S31 | 11,673,263 | 10,794,687 | 193.53 | 92.47% | 30.08 |
| HAN S32 | 12,184,781 | 11,250,926 | 193.6 | 92.34% | 31.36 |
| HAN S33 | 12,035,384 | 11,141,070 | 193.64 | 92.57% | 31.06 |
| HAN S34 | 12,182,139 | 11,325,444 | 193.58 | 92.97% | 31.56 |
| HAN S35 | 11,363,680 | 10,566,389 | 193.52 | 92.98% | 29.44 |
| HAN S36 | 11,237,921 | 10,490,286 | 193.61 | 93.35% | 29.24 |
| HAN S37 | 12,226,645 | 11,329,068 | 193.69 | 92.66% | 31.59 |
| HAN S38 | 12,285,294 | 11,380,424 | 193.61 | 92.63% | 31.72 |
| HAN S39 | 11,110,411 | 10,286,962 | 193.99 | 92.59% | 28.73 |
| HAN S40 | 11,959,742 | 11,038,841 | 193.44 | 92.30% | 30.74 |

Table S2 | The sample size information of applied populations in the 1KG dataset.

| Dataset | POP | Sample size | With RNA data | DNA-RNA mismatch filtered | Batch effect filtered |
| --- | --- | --- | --- | --- | --- |
| KGP phase III | GBR | 94 | 86 | 86 | 68 |
|  | CEU | 91 | 89 | 89 | 70 |
|  | FIN | 95 | 92 | 92 | 71 |
|  | TSI | 93 | 91 | 91 | 72 |
|  | YRI | 89 | 87 | 87 | 69 |

Note: “Sample Size” referred to the sample size of each population with DNA data; “With RNA data” referred to the sample size with RNA data; “DNA-RNA mismatch filtered” referred to the sample size filtered with DNA-RNA mismatch; “Batch effect filtered” was referred to the sample size filtered with batch effects.

Table S3 | The properties of ASE detection at XJU individual level.

| Sample ID | HDS-ASE  number | All ASE  number | Heterozygotes  number | HDS-ASE/ All ASE  Ratio |
| --- | --- | --- | --- | --- |
| XJU S1 | 16 | 222 | 2146451 | 7.21% |
| XJU S2 | 39 | 247 | 2125441 | 15.79% |
| XJU S3 | 30 | 223 | 2159570 | 13.45% |
| XJU S4 | 15 | 218 | 2158861 | 6.88% |
| XJU S5 | 39 | 339 | 2144051 | 11.50% |
| XJU S6 | 23 | 248 | 2162893 | 9.27% |
| XJU S7 | 62 | 373 | 2137187 | 16.62% |
| XJU S8 | 28 | 215 | 2055153 | 13.02% |
| XJU S9 | 36 | 279 | 2132547 | 12.90% |
| XJU S10 | 35 | 274 | 2144228 | 12.77% |
| XJU S11 | 47 | 267 | 2080054 | 17.60% |
| XJU S12 | 38 | 348 | 2134864 | 10.92% |
| XJU S13 | 15 | 333 | 2167089 | 4.50% |
| XJU S14 | 34 | 199 | 2148025 | 17.09% |
| XJU S15 | 22 | 242 | 2148237 | 9.09% |
| XJU S16 | 27 | 350 | 2136174 | 7.71% |
| XJU S17 | 37 | 248 | 2119945 | 14.92% |
| XJU S18 | 22 | 360 | 2157407 | 6.11% |
| XJU S19 | 27 | 459 | 2109726 | 5.88% |
| XJU S20 | 28 | 415 | 2157791 | 6.75% |
| XJU S21 | 35 | 285 | 2165679 | 12.28% |
| XJU S22 | 31 | 326 | 2138578 | 9.51% |
| XJU S23 | 37 | 248 | 2165369 | 14.92% |
| XJU S24 | 20 | 267 | 2074219 | 7.49% |
| XJU S25 | 43 | 242 | 2186172 | 17.77% |
| XJU S26 | 28 | 248 | 2158686 | 11.29% |
| XJU S27 | 27 | 270 | 2098267 | 10.00% |
| XJU S28 | 24 | 203 | 2150837 | 11.82% |
| XJU S29 | 31 | 227 | 2118358 | 13.66% |
| XJU S30 | 20 | 261 | 2049380 | 7.66% |
| XJU S31 | 21 | 203 | 2132916 | 10.34% |
| XJU S32 | 27 | 247 | 2168593 | 10.93% |
| XJU S33 | 27 | 262 | 2143884 | 10.31% |
| XJU S34 | 30 | 219 | 2135292 | 13.70% |
| XJU S35 | 40 | 324 | 2125539 | 12.35% |
| XJU S36 | 33 | 335 | 2115011 | 9.85% |
| XJU S37 | 42 | 344 | 2075149 | 12.21% |
| XJU S38 | 45 | 355 | 2146273 | 12.68% |
| XJU S39 | 17 | 314 | 2145706 | 5.41% |
| XJU S40 | 38 | 471 | 2135943 | 8.07% |
| XJU S41 | 38 | 245 | 2124410 | 15.51% |
| XJU S42 | 21 | 283 | 2170788 | 7.42% |
| XJU S43 | 24 | 282 | 2139529 | 8.51% |
| XJU S44 | 32 | 348 | 2143152 | 9.20% |
| XJU S45 | 34 | 395 | 1979578 | 8.61% |
| XJU S46 | 31 | 240 | 2154724 | 12.92% |
| XJU S47 | 18 | 254 | 2139392 | 7.09% |
| XJU S48 | 40 | 278 | 2109189 | 14.39% |
| XJU S49 | 26 | 289 | 2164845 | 9.00% |
| XJU S50 | 24 | 259 | 2144262 | 9.27% |
| XJU S51 | 28 | 300 | 2134755 | 9.33% |
| XJU S52 | 20 | 363 | 2139450 | 5.51% |
| XJU S53 | 25 | 354 | 2159818 | 7.06% |
| XJU S54 | 24 | 389 | 2146984 | 6.17% |
| XJU S55 | 18 | 310 | 2139205 | 5.81% |
| XJU S56 | 41 | 296 | 2135845 | 13.85% |
| XJU S57 | 16 | 143 | 1756642 | 11.19% |
| XJU S58 | 20 | 269 | 2143892 | 7.43% |
| XJU S59 | 28 | 298 | 2145986 | 9.40% |
| XJU S60 | 48 | 285 | 2107604 | 16.84% |
| XJU S61 | 57 | 394 | 2119217 | 14.47% |
| XJU S62 | 27 | 229 | 2076305 | 11.79% |
| XJU S63 | 48 | 459 | 2168942 | 10.46% |
| XJU S64 | 37 | 287 | 2142270 | 12.89% |
| XJU S65 | 50 | 302 | 2152508 | 16.56% |
| XJU S66 | 53 | 296 | 2133507 | 17.91% |
| XJU S67 | 29 | 305 | 2138221 | 9.51% |
| XJU S68 | 31 | 355 | 2145471 | 8.73% |
| XJU S69 | 47 | 354 | 2131431 | 13.28% |
| XJU S70 | 65 | 461 | 2172490 | 14.10% |
| XJU S71 | 41 | 310 | 2133975 | 13.23% |
| XJU S72 | 44 | 326 | 2124870 | 13.50% |
| XJU S73 | 22 | 275 | 2022635 | 8.00% |
| XJU S74 | 18 | 302 | 2126346 | 5.96% |
| XJU S75 | 29 | 202 | 2124986 | 14.36% |
| XJU S76 | 43 | 306 | 2101804 | 14.05% |
| XJU S77 | 23 | 302 | 2157712 | 7.62% |
| XJU S78 | 44 | 323 | 2099249 | 13.62% |
| XJU S79 | 18 | 312 | 2160827 | 5.77% |
| XJU S80 | 19 | 237 | 2150307 | 8.02% |
| XJU S81 | 38 | 286 | 2134084 | 13.29% |
| XJU S82 | 51 | 434 | 2142726 | 11.75% |
| XJU S83 | 30 | 429 | 2105674 | 6.99% |
| XJU S84 | 31 | 239 | 2161056 | 12.97% |
| XJU S85 | 27 | 347 | 2148940 | 7.78% |
| XJU S86 | 25 | 312 | 2044041 | 8.01% |
| XJU S87 | 30 | 217 | 2156604 | 13.82% |
| XJU S88 | 32 | 378 | 2149031 | 8.47% |
| XJU S89 | 32 | 271 | 2140669 | 11.81% |
| XJU S90 | 26 | 325 | 2142064 | 8.00% |

Table S4 | The 31 ancestral-like genes were eQTLAS-genes in XJU.

| eQTAS-genes correlated Eastern-like segments | eQTAS-genes correlated Western-like segments |
| --- | --- |
| *HEXDC* | *LPIN1* |
| *UTS2* | *RP1-283E3.4* |
| *NEDD4* | *RP11-326C3.13* |
| *LILRB2* | ***POLR2B*** |
| *KIR2DS4* | *GNLY* |
| *MZT2A* | *RP11-274B18.2* |
| *SPHK1* | *LRRC8B* |
| *PTPRN2* | *PDXDC1* |
| *UAP1L1* | *PGM5* |
| *CD151* | *RP11-479G22.8* |
| *GCC2* | *AFAP1* |
| *NARFL* | *CCDC163P* |
| ***PKP4*** | *RRP7A* |
| *CTSW* | *CTD-2547E10.2* |
| ***HLA-DRB6*** | *LRRC37A* |
| *-* | *SIGLEC14* |

Note: The genes in bold indicated the genes were not identified as HDS-eGenes or/ and HDS-aseGenes. The ‘-’ indicated empty in the table.

Table S5 | The 144 collected T2D-related studies applied in our T2D enrichment analysis.

Note: this table was in the separate tsv file

# Reference

1. Patterson N, Price AL, Reich D. Population Structure and Eigenanalysis. *PLOS Genetics*. 2006; **2**(12): e190. doi: 10.1371/journal.pgen.0020190

2. Price AL, Patterson NJ, Plenge RM *et al.* Principal components analysis corrects for stratification in genome-wide association studies. *Nature Genetics*. 2006; **38**(8): 904-909. doi: 10.1038/ng1847

3. Pan Y, Zhang C, Lu Y *et al.* Genomic diversity and post-admixture adaptation in the Uyghurs. *National Science Review*. 2021. doi: 10.1093/nsr/nwab124

4. Feng Q, Lu Y, Ni X *et al.* Genetic History of Xinjiang's Uyghurs Suggests Bronze Age Multiple-Way Contacts in Eurasia. *Mol Biol Evol*. 2017; **34**(10): 2572-2582. doi: 10.1093/molbev/msx177

5. Pruitt KD, Harrow J, Harte RA *et al.* The consensus coding sequence (CCDS) project: Identifying a common protein-coding gene set for the human and mouse genomes. *Genome Res*. 2009; **19**(7): 1316-1323. doi: 10.1101/gr.080531.108

6. Dobin A, Davis CA, Schlesinger F *et al.* STAR: ultrafast universal RNA-seq aligner. *Bioinformatics*. 2013; **29**(1): 15-21. doi: 10.1093/bioinformatics/bts635

7. Li B, Dewey CN. RSEM: accurate transcript quantification from RNA-Seq data with or without a reference genome. *BMC Bioinformatics*. 2011; **12**(1): 323. doi: 10.1186/1471-2105-12-323

8. Shen S, Park JW, Lu ZX *et al.* rMATS: robust and flexible detection of differential alternative splicing from replicate RNA-Seq data. *Proc Natl Acad Sci U S A*. 2014; **111**(51): E5593-5601. doi: 10.1073/pnas.1419161111

9. BN H, P D, J M. A Flexible and Accurate Genotype Imputation Method for the Next Generation of Genome-Wide Association Studies. *PLoS Genet* 2009; **5(6): e1000529**. doi: <https://doi.org/10.1371/journal.pgen.1000529>

10. Purcell S, Neale B, Todd-Brown K *et al.* PLINK: a tool set for whole-genome association and population-based linkage analyses. *Am J Hum Genet*. 2007; **81**(3): 559-575. doi: 10.1086/519795

11. Gao Y, Zhang C, Yuan L *et al.* PGG.Han: the Han Chinese genome database and analysis platform. *Nucleic Acids Res*. 2020; **48**(D1): D971-D976. doi: 10.1093/nar/gkz829

12. Lu D, Lou H, Yuan K *et al.* Ancestral Origins and Genetic History of Tibetan Highlanders. *Am J Hum Genet*. 2016; **99**(3): 580-594. doi: 10.1016/j.ajhg.2016.07.002

13. Deng L, Zhang C, Yuan K *et al.* Prioritizing natural-selection signals from the deep-sequencing genomic data suggests multi-variant adaptation in Tibetan highlanders. *National Science Review*. 2019; **6**(6): 1201-1222. doi: 10.1093/nsr/nwz108

14. Ma X, Yang W, Gao Y *et al.* Genetic Origins and Sex-Biased Admixture of the Huis. *Mol Biol Evol*. 2021; **38**(9): 3804-3819. doi: 10.1093/molbev/msab158

15. Zhang C, Gao Y, Ning Z *et al.* PGG.SNV: understanding the evolutionary and medical implications of human single nucleotide variations in diverse populations. *Genome biology*. 2019; **20**(1): 215-215. doi: 10.1186/s13059-019-1838-5

16. Patterson N, Price AL, Reich D. Population structure and eigenanalysis. *PLoS Genet*. 2006; **2**(12): e190. doi: 10.1371/journal.pgen.0020190

17. Stegle O, Parts L, Durbin R *et al.* A Bayesian framework to account for complex non-genetic factors in gene expression levels greatly increases power in eQTL studies. *PLoS Comput Biol*. 2010; **6**(5): e1000770. doi: 10.1371/journal.pcbi.1000770

18. Shabalin AA. Matrix eQTL: ultra fast eQTL analysis via large matrix operations. *Bioinformatics*. 2012; **28**(10): 1353-1358. doi: 10.1093/bioinformatics/bts163

19. Xu S, Jin L. A genome-wide analysis of admixture in Uyghurs and a high-density admixture map for disease-gene discovery. *Am J Hum Genet*. 2008; **83**(3): 322-336. doi: 10.1016/j.ajhg.2008.08.001

20. Xu S, Huang W, Qian J *et al.* Analysis of genomic admixture in Uyghur and its implication in mapping strategy. *Am J Hum Genet*. 2008; **82**(4): 883-894. doi: 10.1016/j.ajhg.2008.01.017

21. Genomes Project C, Auton A, Brooks LD *et al.* A global reference for human genetic variation. *Nature*. 2015; **526**(7571): 68-74. doi: 10.1038/nature15393

22. Lappalainen T, Sammeth M, Friedlander MR *et al.* Transcriptome and genome sequencing uncovers functional variation in humans. *Nature*. 2013; **501**(7468): 506-511. doi: 10.1038/nature12531

23. Sudmant PH, Rausch T, Gardner EJ *et al.* An integrated map of structural variation in 2,504 human genomes. *Nature*. 2015; **526**(7571): 75-81. doi: 10.1038/nature15394

24. Danecek P, Auton A, Abecasis G *et al.* The variant call format and VCFtools. *Bioinformatics*. 2011; **27**(15): 2156-2158. doi: 10.1093/bioinformatics/btr330

25. Zerbino DR, Wilder SP, Johnson N *et al.* The Ensembl Regulatory Build. *Genome Biology*. 2015; **16**(1): 56. doi: 10.1186/s13059-015-0621-5

26. Yu G, Wang LG, Han Y *et al.* clusterProfiler: an R package for comparing biological themes among gene clusters. *OMICS*. 2012; **16**(5): 284-287. doi: 10.1089/omi.2011.0118

27. Liberzon A, Birger C, Thorvaldsdottir H *et al.* The Molecular Signatures Database (MSigDB) hallmark gene set collection. *Cell Syst*. 2015; **1**(6): 417-425. doi: 10.1016/j.cels.2015.12.004

28. Liberzon A, Subramanian A, Pinchback R *et al.* Molecular signatures database (MSigDB) 3.0. *Bioinformatics*. 2011; **27**(12): 1739-1740. doi: 10.1093/bioinformatics/btr260

29. Subramanian A, Tamayo P, Mootha VK *et al.* Gene set enrichment analysis: A knowledge-based approach for interpreting genome-wide expression profiles. *Proceedings of the National Academy of Sciences*. 2005; **102**(43): 15545-15550. doi: 10.1073/pnas.0506580102

30. Hindorff LA, Sethupathy P, Junkins HA *et al.* Potential etiologic and functional implications of genome-wide association loci for human diseases and traits. *Proc Natl Acad Sci U S A*. 2009; **106**(23): 9362-9367. doi: 10.1073/pnas.0903103106

31. Nédélec Y, Sanz J, Baharian G *et al.* Genetic Ancestry and Natural Selection Drive Population Differences in Immune Responses to Pathogens. *Cell*. 2016; **167**(3): 657-669.e621. doi: 10.1016/j.cell.2016.09.025

32. Buniello A, MacArthur JAL, Cerezo M *et al.* The NHGRI-EBI GWAS Catalog of published genome-wide association studies, targeted arrays and summary statistics 2019. *Nucleic Acids Res*. 2019; **47**(D1): D1005-d1012. doi: 10.1093/nar/gky1120

33. Prokopenko I, McCarthy MI, Lindgren CM. Type 2 diabetes: new genes, new understanding. *Trends in Genetics*. 2008; **24**(12): 613-621. doi: <https://doi.org/10.1016/j.tig.2008.09.004>

34. Castel SE, Mohammadi P, Chung WK *et al.* Rare variant phasing and haplotypic expression from RNA sequencing with phASER. *Nature Communications*. 2016; **7**. doi: 10.1038/ncomms12817

35. Castel SE, Levy-Moonshine A, Mohammadii P *et al.* Tools and best practices for data processing in allelic expression analysis. *Genome Biol*. 2015; **16**: 12. doi: 10.1186/s13059-015-0762-6

36. Battle A, Mostafavi S, Zhu X *et al.* Characterizing the genetic basis of transcriptome diversity through RNA-sequencing of 922 individuals. *Genome Res*. 2014; **24**(1): 14-24. doi: 10.1101/gr.155192.113

37. Dias-Alves T, Mairal J, Blum MGB. Loter: A Software Package to Infer Local Ancestry for a Wide Range of Species. *Mol Biol Evol*. 2018; **35**(9): 2318-2326. doi: 10.1093/molbev/msy126

38. Xu S, Jin W, Jin L. Haplotype-sharing analysis showing Uyghurs are unlikely genetic donors. *Mol Biol Evol*. 2009; **26**(10): 2197-2206. doi: 10.1093/molbev/msp130

39. Sudmant PH, Rausch T, Gardner EJ *et al.* An integrated map of structural variation in 2,504 human genomes. *Nature*. 2015; **526**(7571): 75-81. doi: 10.1038/nature15394

40. Stegle O, Parts L, Piipari M *et al.* Using probabilistic estimation of expression residuals (PEER) to obtain increased power and interpretability of gene expression analyses. *Nature Protocols*. 2012; **7**(3): 500-507. doi: 10.1038/nprot.2011.457

41. Rishishwar L, Conley AB, Wigington CH *et al.* Ancestry, admixture and fitness in Colombian genomes. *Sci Rep*. 2015; **5**(1): 12376. doi: 10.1038/srep12376
